# Clinical outcomes and peripheral tissue oxygen saturation monitoring of the knee region by near-infrared spectroscopy in circulatory shock: a prospective observational cohort study

**DOI:** 10.1186/s13054-025-05363-1

**Published:** 2025-03-19

**Authors:** Elina Varis, Maria Heliste, Johanna Hästbacka, Suvi T. Vaara, Markus B. Skrifvars, Ville Pettilä, Mitja Lääperi, Anne Kuitunen, Annukka Vahtera, Erika Wilkman

**Affiliations:** 1 Helsinki University Hospital (Perioperative and Intensive Care), University of Helsinki, Helsinki, Finland; 2 Faculty of Medicine and Health Technology, Tampere University Hospital, Wellbeing Services County of Pirkanmaa, and Tampere University, Tampere, Finland; 3 Lääperi Statistical Consulting, Espoo, Finland

**Keywords:** Circulatory shock, Tissue perfusion, Tissue oxygen saturation, Near-infrared spectroscopy

## Abstract

**Background:**

In circulatory shock, tissue hypoperfusion leads to adverse outcomes. We hypothesized that peripheral tissue oxygen saturation (StO_2_), measured with near-infrared spectroscopy (NIRS), could provide a non-invasive method for assessing tissue hypoperfusion and predicting pending organ dysfunction and mortality.

**Methods:**

ASSESS-SHOCK was a prospective, observational study enrolling circulatory shock patients from April 2019 to May 2023 in three intensive care units (ICU). Adult patients fulfilling the criteria for circulatory shock within 24 h of ICU admission were eligible. Patients underwent continuous 48 h StO_2_ (INVOS™) monitoring of the knee region. To express the burden of tissue hypoperfusion we calculated mean StO_2_ and areas below predefined StO_2_ thresholds. The primary outcome, change in Sequential Organ Failure Assessment (SOFA) score, was dichotomized to improvement or non-improvement in SOFA score from enrollment to day 7 or ICU discharge. Death within 7 days was considered as SOFA non-improvement. 90-day mortality was among the secondary outcomes.

**Results:**

We included 256 patients. Due to several reasons, including the COVID-19 pandemic, the patient sample was not consecutive. The median of 48-h mean StO_2_ was 68.3% (interquartile range [IQR] 57.5–74.1) in SOFA-improvers (n = 171), compared to 63.5% (IQR 52.7–70.8, *p* = 0.020) in non-improvers (n = 85), and 68.7% (IQR 58.2–74.5) in 90-day survivors, versus 60.9% (IQR 49.5–67.1, *p* < 0.001) in non-survivors. There were no statistically significant differences in the areas below predefined StO_2_ thresholds between the SOFA-improvers and non-improvers but all the areas were larger in 90-day non-survivors. The 90-day mortality was 27.0% (n = 69). In multivariable analyses 48-h mean StO_2_ was associated with 90-day mortality (Odds ratio [OR] 0.97, 95% confidence interval [CI 95%] 0.94–1.00, *p* = 0.047) but not with SOFA change. The association with mortality was, however, no longer significant in multivariable analyses after exclusion of the last 6 hours of StO_2_ registration in the patients (n = 29) who died during the 48 h registration (OR 0.97, CI 95% 0.94–1.00, *p* = 0.062).

**Conclusions:**

Lower peripheral StO_2_ was associated with 90-day mortality in critically ill patients with circulatory shock but not with persisting or worsening organ dysfunction. NIRS shows promise as a non-invasive monitor of tissue perfusion in circulatory shock.

*Trial registration*: ClinicalTrials.gov Identifier: NCT03814564, registered 15 January 2019.

**Supplementary Information:**

The online version contains supplementary material available at 10.1186/s13054-025-05363-1.

## Background

In management of circulatory shock, the main goal of hemodynamic resuscitation is to secure adequate tissue perfusion. As an indirect marker of tissue perfusion, near infrared spectroscopy (NIRS) offers a non-invasive, continuous method to monitor tissue oxygen saturation (StO_2_) in both central and peripheral tissues [1]. The rationale for monitoring peripheral tissues, such as lower limbs, is that due to compensatory centralization of circulation in shock, these tissues are the first to manifest and last to recover from hypoperfusion [2]. Indeed, mottling of the skin and the StO_2_ of the knee region predicted mortality in septic shock patients in the landmark studies on peripheral perfusion by Ait-Oufella and coworkers [3, 4]. Currently, the perioperative use of cerebral StO_2_ monitoring is routinely recommended to prevent complications mainly in cardiac surgery [5]. The role of peripheral tissue StO_2_ monitoring, however, is less established [6]. In intensive care medicine, StO_2_ monitoring has mainly been studied in predicting different clinical outcomes [7–10].

In the ASSESS-SHOCK study, we aimed to explore the utility of NIRS monitoring in circulatory shock patients in the intensive care unit (ICU). We hypothesized that monitoring peripheral StO_2_ could provide a continuous and objective method to identify patients at risk for persisting or worsening organ dysfunction. Our primary objective was to evaluate whether knee StO_2_ associates with the evolution of organ dysfunction assessed by a change in Sequential Organ Failure Assessment (SOFA) score. Secondary objectives were to explore the association of knee StO_2_ with 28- and 90-day mortality, and feasibility of NIRS monitoring in the ICU.

## Methods

ASSESS-SHOCK study was a prospective, observational, investigator initiated, multicenter study that included 325 patients with circulatory shock between April 2019 and May 2023 at three intensive care units at Helsinki University Hospital and Tampere University Hospital. A sub-cohort of 256 patients underwent NIRS monitoring for 48 h.

The ethical principles outlined by the Declaration of Helsinki, the Good Clinical Practice guidelines, and the local regulatory statements guided the management of the study. The ethics committee of Helsinki University Hospital (HUS/3631/2017) approved the study protocol that we pre-registered at ClinicalTrials.gov (NCT03814564). Due to the emergency nature and the severity of disease in the target population, and observational nature of the study, we used a deferred informed consent policy. Patients lacking consent were withdrawn from the analyses. The Strengthening the Reporting of Observational Studies in Epidemiology (the STROBE) Statement checklist [11] served in writing the study report.

### Inclusion criteria

We included adult (≥ 18 years of age) critically ill patients who fulfilled the criteria of circulatory shock within 24 h of ICU admission. We included patients within 4 h of initiation of vasopressor treatment in the ICU. We defined circulatory shock [12, 13] as need for vasopressor treatment to maintain mean arterial pressure (MAP) of at least 65 mmHg despite one liter of crystalloid resuscitation fluids AND either evidence of tissue hypoperfusion (presence of any of the following: blood lactate ≥ 2 mmol/L; mottling score ≥ 2 [3]; base excess [BE] ≤ -5 mEq/L; capillary refill time [CRT] ≥ 2 s; cool periphery beyond elbows or knees bilaterally; or altered mentation) OR diagnosed/suspected infection with antibiotic treatment. The study personnel, or if unavailable, the ICU nurses and clinicians, screened patients for eligibility.

### Exclusion criteria

We excluded pregnant patients, out of hospital cardiac arrest patients, terminally ill patients or patients who were not considered for full intensive care support, planned postoperative admissions, patients on extracorporeal membrane oxygenation, patients who were likely transferred to ward within 24 h, and patients who had vasopressor treatment initiated over 24 h before or after ICU admission. Patients with tissue defects precluding NIRS monitoring were not included in the NIRS sub-cohort.

### Outcome measures

The primary outcome measure of the study was change in severity of organ dysfunction measured by the change in the total SOFA score from day 1 to day 7 or discharge from ICU. We dichotomized patients into “SOFA improvers” and "SOFA non-improvers”. We categorized those whose SOFA score decreased and who survived to day 7 as “SOFA improvers”, and those whose SOFA score increased, remained unchanged or who died during the 7 day follow-up in ICU, as “SOFA non-improvers” [14]. Secondary outcome measures were 28 and 90-day mortality; and feasibility of INVOS™ NIRS StO_2_ monitoring.

### Hemodynamic resuscitation

Attending ICU clinicians were responsible for the hemodynamic resuscitation according to current international guidelines and local standard operating procedures (SOP) [15, 16]. Briefly, the local SOPs included an initial fluid resuscitation of 20–30 ml/kg of crystalloid solution and early norepinephrine infusion. Repeated evaluation of tissue perfusion, blood lactate and fluid responsiveness guided further fluid administration. The initial MAP target was ≥ 65 mmHg.

### NIRS monitoring and feasibility

We used INVOS™ 5100 regional oximeters (Medtronic, Minneapolis, MN, USA) and bilateral sensors at the suprapatellar knee region and the forehead (INVOS™ Cerebral/Somatic Oximetry Adult Sensor) for continuous StO_2_ registration during the first 48 h after study enrollment. We recorded StO_2_ data at 60 s intervals. The StO_2_ monitors were not visible to the treating ICU nurses and physicians, and StO_2_ registrations were therefore not used for making clinical decisions. We used INVOS™ Analytics Tool (Version 1.2, Medtronic, Minneapolis, USA) to process and save the data for further analysis.

We collected data on feasibility or issues in NIRS monitoring every 8 h. These data were reported by the ICU nurses and ICU physicians using a scale ranging from 0 to 5 points to evaluate whether NIRS monitoring interfered with five aspects of clinical care in the intensive care unit: nursing care, patient positioning, imaging, clinical examination, or procedures performed by the attending physician (0 = not at all, 5 = very much).

### Hemodynamic monitoring

We continuously monitored heart rate, electrocardiography (ECG), and arterial blood pressure invasively with an arterial cannula. We analyzed arterial blood gases and lactate every two hours for the first 48 h using point-of-care devices. Attending ICU physicians performed central venous catheterization as needed, and transthoracic echocardiogram at study enrolment (± 4 h) and at 24 h (± 4 h). ICU nurses assessed peripheral tissue perfusion hourly for the first 48 h by measuring peripheral skin temperature with skin thermometer probes over the brachioradial muscle, CRT (seconds) and mottling score (on a scale from 0 to 5) [3].

### Data collection and handling

We collected and validated routine data, such as demographics, diagnoses (ICD-10), illness severity scores, physiologic measures, and outcome prospectively for the Finnish intensive care quality database (Finnish Intensive Care Consortium database, Tietoevry, Espoo, Finland). Additionally, we manually collected chronic illnesses and medications prior to ICU admission, details of the acute illness, hemodynamic variables, details of medication, fluid balance and specific ICU interventions as well as daily SOFA scores from the electronic patient records in an electronic case report form during the first seven days in the ICU or until the end of ICU stay, which ever came first. We defined sepsis and septic shock according to Sepsis-3 definition [17], acute kidney injury (AKI) according to the Kidney Disease Improving Global Outcomes [18], and Chronic Kidney Disease as glomerular filtration rate < 60 ml/minute/1.73 m^2^ or chronic dialysis at least one week prior to ICU admission.

### Statistical methods

We present data as medians with interquartile ranges (IQR) or counts with percentages as appropriate. We first summarized all continuous clinical and StO_2_ data as arithmetic, time-weighted means for each patient, and then as medians for different patient subpopulations and preplanned time frames (0–24 and 0–48 h). Post hoc*,* we also analyzed data separately for the first six hours. In addition, we calculated the area under the curve (Area under threshold, AUT; StO_2_ [%] ✕ time [minutes], Additional file 1; electronic supplementary material [ESM] Figure S1) below three prespecified StO_2_ thresholds (60%, 50% and 40%) [19, 20] and the aggregate time of the StO_2_ signal below these thresholds. Post hoc*,* we also calculated the area under the curve and time above StO_2_ 80% (area over threshold, AOT) [14]. We calculated all the AUTs, AOTs and times with and without adjustment for the duration of StO_2_ monitoring. As additional analyses, to ensure the inclusion of StO_2_ values only prior to and not simultaneously with the endpoint, we calculated post hoc the 48-h mean StO_2_ excluding the last 6 h of the registration in patients who died during the 48-h StO_2_ registration period. We also calculated the mean and median StO_2_ for hours 1, 12, 24 and the last observation hour to explore the association of early changes in StO_2_ over time (delta) with outcome. We calculated all the StO_2_ based parameters as a mean of the bilateral registrations. We first calculated a mean feasibility score for each patient and then a median for the whole cohort. We predefined NIRS monitoring feasible if the median score was below 3.0. We also calculated the median duration of monitoring and proportion of the missing data.

We used the Mann–Whitney U test and the Fisher´s exact test for difference testing between groups, as appropriate. We used forward conditional multivariate logistic regression analyses in which we included variables with *p* < 0.1 in univariable analyses to determine crude and adjusted risk for persisting or worsening organ dysfunction and 90-day mortality. We deleted cases with missing data listwise and we did not impute missing data. We further assessed predictive properties by calculating area under the receiver operating characteristics curve (AUROC) with 95% confidence intervals (CI 95%). We modeled the behavior of the mean peripheral StO_2_ during the 48-h observation using linear mixed models with random intercepts to account for the repeated measurements. We modeled the change over time non-linearly using restricted cubic splines with 5 knots. We included interaction between the time and outcome. We calculated the adjusted bootstrap percentile intervals confidence intervals using 1000 bootstrap samples. We considered *p*-values below 0.05 significant. We used Excel (version 16.16.27), IBM SPSS Statistics (version 28.0.0.0) and NCSS 2022 Statistical Software (2022, NCSS, LLC. Kaysville, Utah, USA, ncss.com/software/ncss) and R software (version 4.2.2 with packages lme4, rms, and ggplot2) for the analyses.

## Results

### Study population

We enrolled 353 circulatory shock patients fulfilling the inclusion criteria, and none of the exclusion criteria, during the study period from April 2019 to May 2023. We excluded 28 patients due to lack of informed consent, and 69 patients were included after completion of the NIRS sub-cohort and therefore did not undergo NIRS monitoring. Thus, we included 256 patients in the analyses (ESM Figure S2).

The median follow-up time within 90-days was 90.0 (IQR 24.5; 90.0) days. The median age of the study patients was 67.0 (IQR 57.0; 73.0) years and 59.2% were male. Distributive shock was the most frequent type of shock (76%), followed by hypovolemic shock (18%) and cardiogenic shock (4%). A total of 206 patients (81%) had sepsis and 151 (59%) had septic shock at study enrollment [17]. Table 1 and S1 (ESM) present the patient characteristics in detail. The median delay from ICU admission to study enrollment was 2.2 (IQR 1.4; 3.1) hours and the median duration of the preceding hospital admission 15.8 (IQR 6.7; 32.0) hours. Patients had a median of 2 (IQR 2; 3) signs of tissue hypoperfusion at enrollment (Table S1 and Figs. S3a and b, ESM).

**Table 1:** Patient characteristics

	Missing	All patients	Organ dysfunction			90-day mortality		
			SOFA-improvers	SOFA-non-improvers	*p*	Survivors	Non-survivors	*p*
	n	n = 256	n = 171	n = 85		n = 187	n = 69	
Age (years)	0	67.0 (57.0; 73.0)	68.0 (57.0; 74.0)	65 (58.0; 71.5)	0.478	66.0 (55.0; 73.0)	68.0 (61.0; 75.0)	0.054
Gender (female)	0	103 (40.2)	64 (37.4)	39 (45.9)	0.224	74 (39.6)	29 (42.0)	0.774
Co-morbidity								
Total Charlson Comorbidity Index score	0	4.0 (2.0; 6.0)	4.0 (2.0; 6.0)	4.0 (2.5; 6.0)	0.782	4.0 (2.0; 5.0)	5.0 (3.0; 7.0)	0.002
Diabetes mellitus	0	91 (35.5)	58 (33.9)	33 (38.8)	0.489	65 (34.8)	26 (37.7)	0.662
Peripheral or cerebral arterial disease	0	61 (23.8)	40 (23.4)	21 (24.7)	0.876	41 (21.9)	20 (29.0)	0.25
Coronary artery disease	0	35 (13.7)	26 (15.2)	9 (10.6)	0.342	25 (13.4)	10 (14.5)	0.839
Hypertension	0	162 (63.3)	110 (64.3)	52 (61.2)	0.68	115 (61.5)	47 (68.1)	0.382
Chronic kidney disease	0	32 (12.5)	18 (10.5)	14 (16.5)	0.228	19 (10.2)	13 (18.8)	0.087
Chronic heart failure	0	22 (8.6)	18 (10.5)	4 (4.7)	0.156	17 (9.1)	5 (7.2)	0.803
Smoking (ever)	0	119 (46.5)	66 (38.6)	53 (62.4)	< 0.001	77 (41.2)	42 (60.9)	0.007
APACHE IV diagnostic group	0							
Cardiovascular (non-operative)		64 (25.0)	43 (25.1)	21 (24.7)	0.762	49 (26.2)	15 (21.7)	0.829
Cardiovascular (operative)		24 (9.4)	13 (7.6)	11 (12.9)		17 (9.1)	7 (10.1)	
Respiratory tract (non-operative)		19 (7.4)	15 (8.8)	4 (4.7)		13 (7.0)	6 (8.7)	
Gastrointestinal tract (non-operative)		19 (7.4)	12 (7.0)	7 (8.2)		13 (7.0)	6 (8.7)	
Gastrointestinal tract (operative)		84 (32.8)	60 (35.1)	24 (28.2)		59 (31.6)	25 (36.2)	
Other (< 5% each)		65 (25.4)	28 (16.4)	18 (21.2)		36 (19.3)	10 (14.5)	
Invasive mechanical ventilation	0	200 (78.1)	134 (78.4)	66 (77.6)	1	137 (73.3)	63 (91.3)	0.002
Norepinephrine dose at enrolment	0	0.22 (0.12; 0.38)	0.20 (0.11; 0.33)	0.25 (0.13; 0.48)	0.044	0.20 (0.11; 0.33)	0.28 (0.13; 0.55)	0.003
Lactate at enrolment	24	2.9 (1.6; 5.2)	2.4 (1.5; 4.7)	3.4 (1.9; 5.9)	0.03	2.2 (1.5; 4.0)	4.8 (2.9; 7.3)	< 0.001
Dominant shock type	3							
Distributive		194 (76.7)	131 (76.6)	63 (76.8)	0.954	137 (74.1)	57 (83.8)	0.339
Cardiogenic		11 (4.3)	8 (4.7)	3 (3.7)		10 (5.4)	1 (1.5)	
Hypovolemic		46 (18.0)	30 (17.5)	16 (19.5)		36 (19.5)	10 (14.7)	
Obstructive		2 (0.8)	2 (1.2)	0		2 (1.1)	0	
Sepsis diagnosis at enrolment	0	206 (80.5)	139 (81.3)	67 (78.8)	0.738	148 (79.1)	58 (84.1)	0.478
Septic shock diagnosis at enrolment	0	151 (59.0)	98 (57.3)	53 (62.4)	0.5	100 (53.5)	51 (73.9)	0.004
AKI during days 1–7	0	167 (65.2)	103 (60.2)	64 (75.3)	0.018	106 (56.7)	61 (88.4)	< 0.001
RRT (CRRT or IHD or both) DURING days 1–7	0	80 (31.3)	39 (22.8)	41 (48.2)	< 0.001	45 (24.1)	35 (50.7)	< 0.001
SOFA 24 h	2	10.0 (8.0; 13.0)	9.0 (8.0; 12.0)	10.0 (7.0; 13.0)	0.126	9.0 (7.0; 11.0)	13.0 (9.0; 15.0)	< 0.001
Length of ICU stay (days)	0	4.1 (2.1; 7.3)	5.1 (2.9; 8.5)	2.0 (1.3; 4.4)	< 0.001	4.8 (2.3; 7.3)	2.2 (1.5; 7.3)	0.006
ICU mortality	0	50 (19.5)	5 (2.9)	45 (52.9)	< 0.001	0	50 (72.5)	
28-day mortality	0	65 (25.4)	17 (9.9)	48 (56.5)	< 0.001	0	65 (94.2)	
90-day mortality	0	69 (27.0)	19 (11.1)	50 (58.8)	< 0.001	0	69 (100)	

Continuous variables are summarized as medians with interquartile ranges (IQR), categorical variables as counts and percentages

Organ dysfunction was defined as unchanged or worsening SOFA score or death within 7 days in intensive care unit

*SOFA* Sequential organ failure assessment; SOFA-improvers, patients whose SOFA score decreased during the 7 day follow-up; SOFA-non-improvers, patients whose SOFA score stayed the same or increased, or who died during the 7-day follow-up; Chronic Kidney Disease defined as glomerular filtration rate (GFR) < 60 ml/minute/1.73 m^2^ or chronic dialysis at least one week prior to ICU admission; *APACHE* Acute physiology and chronic health evaluation; Sepsis and septic shock defined according to Sepsis-3 [17]; *AKI* Acute kidney injury, defined by Kidney Disease Improving Global Outcomes (KDIGO) Clinical Practice Guidelines [18]; *RRT* Renal replacement therapy; *CRRT* Continuous renal replacement therapy; *IHD* Intermittent hemodialysis; *SAPS II* Simplified acute physiology score; *ICU* Intensive care unit

### Organ dysfunction and mortality

During the first 7 days, SOFA score increased in 31 patients, decreased in 175 patients, and remained unchanged in 50 patients. Furthermore, 40 patients died in the ICU during the first 7 days, 16 patients with increasing, 4 with decreasing, and 18 with unchanged SOFA score. Thus, we categorized a total of 171 (67%) patients as “SOFA-improvers”, since 4 patients died during the first 7 days despite of decreasing SOFA score. Furthermore, 85 (33%) patients were categorized as “SOFA-non-improvers”; 31 with increasing SOFA score, 50 with unchanged SOFA score, and 4 patients who died during the first 7 days in ICU. Median SOFA score for all patients was 10 (IQR 8; 13) at day 1, and 7 (5–11) at day 7 for those alive and still in the ICU (n = 84). Median change in SOFA score was − 2 (IQR − 5; 0) for the whole cohort, − 4 (IQR − 6; − 2) for the SOFA-improvers, and 0 (IQR 0; 1) for the SOFA-non-improvers (Table S1, ESM). The relationship of mean 48-h StO_2_ with SOFA change can be viewed in Fig. S4 (ESM). Median of maximun SOFA score for all patients during the first 7 days was 10 (IQR 8; 13). Of the 256 patients, 167 (65%) patients developed AKI and 80 (31%) received renal replacement therapy during the first 7 days of their ICU stay. All patients received norepinephrine, the median highest dose during the first 48 h being 0.34 (IQR 0.18; 0.64) μg/kg/min. Only three patients (1%) included in the study also received vasopressin. Inotropes were administered to 29 (11%) patients. A total of 194 (76%) of the 256 patients alive and still in the ICU were mechanically ventilated on day 1, and 46 (55%) of the 84 patients on day 7. Overall mortality at 90 days was 27% (n = 69). A total of 29 (42%) of the deaths occurred within the 48-h StO_2_ monitoring period.

### Association of StO_2_ with organ dysfunction and mortality

We present comparisons of illness severity, hemodynamic variables and StO_2_ between SOFA improvers and non-improvers, and 90-day survivors and non-survivors in Table 2, additional parameters and time-intervals in Table S2a and b (ESM), and the AUROC analyses in Tables S3a and b, and Figs. S5a and b (ESM). The 48-h mean StO_2_ was higher in SOFA-improvers (68.3% [IQR 57.5; 74.1], n = 171), than in non-improvers (63.5% [IQR 52.7; 70.8], n = 85), *p* = 0.020. Patients with AKI had similar 48-h mean StO_2_ as the patients without AKI (median 65.3 [IQR 55.6; 72.0] vs. 68.9 [57.8; 75.1] respectively, *p* = 0.060). The 48-h mean StO_2_ was also higher in 90-day survivors (68.7% [IQR 58.2; 74.5]) than in non-survivors (60.9% [IQR 49.5; 67.1], *p* = 0.001). The 29 patients, who died within the 48-h StO_2_ monitoring period were more severely ill compared to patients who died later within 90-days, and a majority (n = 24, 83%), of them died with septic shock and fulminant multiple organ failure. (Table S4 ESM). Of these early deaths, 25 (86%) were preceded a decision to withdraw life sustaining therapy due to treatment failure. The results of univariable analyses were similar when excluding the last 6 h of StO_2_ registration of the patients who died during the StO_2_ registration (Table S5 ESM).

**Table 2:** Results of univariable analyses for organ dysfunction and 90-day mortality

	Missing	Organ dysfunction			90-day mortality		
		SOFA-improvers	SOFA-non-improvers	*p*	Survivors	Non-survivors	*p*
	n	n = 171	n = 85		n = 187	n = 69	
SOFA -score^a^	2	9.0 (8.0; 12.0)	10.0 (7.0; 13.0)	0.126	9.0 (7.0; 11.0)	13.0 (9.0; 15.0)	< 0.001
SAPS II^a^	3	45.0 (37.0; 57.5)	53.5 (39.0; 67.0)	0.009	42.0 (35.0; 53.0	61.5 (51.3; 72.0)	< 0.001
APACHE^a^	3	24.0 (18.0; 29.5)	26.5 (20.0; 34.0)	0.1	23.0 (18.0; 29.0)	29.0 (24.0; 35.0)	< 0.001
Mean arterial pressure (mmHg)^b^	3	74.5 (70.2; 78.2)	69.3 (63.8; 74.0)	< 0.001	73.9 (70.2; 78.0)	68.9 (62.2; 72.8)	< 0.001
Heart rate (bpm)^b^	3	91.1 (79.8; 102.4)	96.4 (81.2; 109.5)	0.094	90.4 (79.1; 101.9)	96.7 (83.6; 109.8)	0.005
Norepinephrine dose (μg/kg/min)^b^	4	0.10 (0.05; 0.24)	0.20 (0.07; 0.42)	0.002	0.10 (0.05; 0.23)	0.25 (0.13; 0.47)	< 0.001
Arterial lactate (mmol/l)^b^	12	1.6 (1.1; 2.4)	3.0 (1.4; 5.8)	< 0.001	1.5 (1.1; 2.3)	4.2 (2.4; 6.5)	< 0.001
Capillary refill time (s)^b^	0	4.5 (2.8; 7.4)	6.3 (3.3; 12.8)	0.006	4.3 (2.8; 7.2)	9.3 (3.9; 14.9)	< 0.001
Mottling score^b^	0	0.0 (0.0; 0.3)	0.1 (0.0; 0.9)	0.023	0.0 (0.0; 0.2)	0.4 (0.0; 1.6)	< 0.001
Peripheral temperature (°C)^b^	1	33.0 (31.8; 34.0)	32.4 (31.0; 33.8)	0.054	32.9 (31.8; 34.0)	32.4 (31.1; 33.7)	0.029
Central to peripheral temperature gradient (°C)^b^	6	4.0 (2.9; 5.2)	4.0 (2.5; 5.6)	0.985	4.0 (2.9; 5.2)	4.0 (2.5; 5.6)	0.909
Mean Peripheral StO_2_ (%)^b^	1	68.3 (57.5; 74.1)	63.5 (52.7; 70.8)	0.02	68.7 (58.2; 74.5)	60.9 (49.5; 67.1)	< 0.001
AUT below StO_2_ thresholds (%min)^b^							
60%	0	1463.0 (39.0; 10,782.0)	1606.3 (81.1; 12,902.5)	0.527	892.0 (6.5; 9135.5)	3963.3 (765.1; 19,927.8)	< 0.001
50%	0	59.3 (0.0; 1811.8)	70.3 (0.0; 3308.5)	0.678	14.0 (0.0; 1502.5)	623.8 (0.0; 6156.0)	0.001
40%	0	0.0 (0.0; 200)	0.0 (0.0; 734.8)	0.245	0.0 (0.0; 160.5)	21.0 (0.0; 993.1)	0.001
AOT above StO_2_ 80% (%min)^b^	0	50.3 (0.0; 1772.8)	0.0 (0.0; 61.8)	< 0.001	50.3 (0.0; 1835.0)	0.0 (0.0; 6.8)	< 0.001
Time below StO_2_ thresholds (min)^b^							
60%	1	221.0 (9.5; 1341.0)	414.5 (18; 1125.1)	0.759	137.0 (3.5; 1215.5)	646.3 (138.1; 1555.0)	0.003
50%	1	5.5 (0.0; 267.5)	8.5 (0.0; 511.13)	0.777	3.0 (0.0; 222.5)	141.0 (0.0; 692.3)	0.002
40%	1	0.0 (0.0; 15.5)	0.0 (0.0; 80.6)	0.408	0.0 (0.0; 9.0)	2.8 (0.0; 151.6)	0.001
Time above StO_2_ 80% (min)^b^	1	15.5 (0.0; 461.0)	0.0 (0.0; 44.3)	< 0.001	15.5 (0.0; 470.0)	0.0 (0.0; 7.1)	< 0.001

All continuous parameters are summarized as medians and interquartile ranges (IQR)

Organ dysfunction was defined as unchanged or worsening SOFA score or death within 7 days in intensive care unit

StO_2_, tissue oxygen saturation; *SOFA* Sequential organ failure assessment; *h* hour; *SOFA* improvers, patients whose SOFA score decreased during the 7 day follow-up; SOFA-non-improvers, patients whose SOFA score stayed the same or increased, or who died during the 7-day follow-up; *SAPS II* Simplified acute physiology score; *APACHE* Acute Physiology and Chronic Health Evaluation II; mmHg, millimeters of mercury; bpm, beats per minute; μg/kg/min, micrograms per kilogram per minute; mmol/l, millimoles per liter; s, seconds; °C, degrees of Celsius; AUT, area under StO_2_ threshold; AOT, area over StO_2_ threshold; min, minutes

^a^0–24 h

^b^48-h mean

A total of 127 (50%) patients had mottling at the knee region during the 48-h follow-up. Median 48-h mean StO_2_ was higher in patients without mottling (70.0% [IQR 60.6; 75.7]) compared to patients with mottling (62.1% [IQR 52.2; 70.0], *p* < 0.001). In both subgroups of patients, with and without mottling, 48-h mean StO_2_ was associated with 90-day mortality but not with organ dysfunction. Median 48-h mean StO_2_ was higher in 90-day survivors compared to 90-day non-survivors in patients without mottling (70.7% [IQR 61.5; 76.3] vs. 63.9% [IQR 53.8; 70.2] *p* = 0.017) as well as in patients with mottling (64.3% [IQR 55.3; 71.4] vs. 57.1% [IQR 48.4; 66.4], *p* = 0.021).

**Fig. 1:**
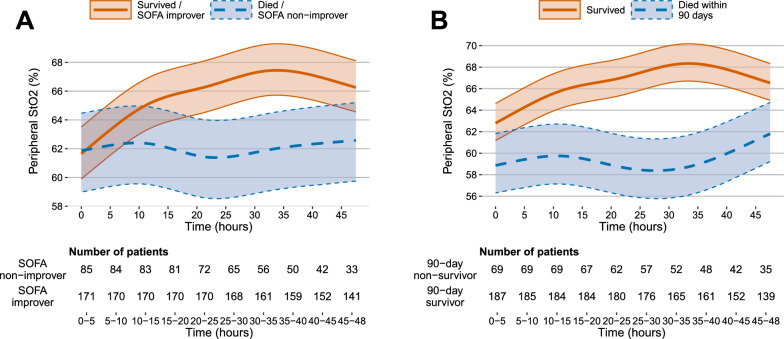
Mean StO_2_ and organ dysfunction and mean StO_2_ and 90-day mortality estimated using linear mixed models. Time was modeled using restricted cubic splines with 5 knots and interaction with organ dysfunction (**a**) or 90-day mortality (**b**). The shaded areas represent the 95% confidence interval. SOFA-improvers were patients, whose SOFA score decreased during the 7 day follow-up, and SOFA-non-improvers patients, whose SOFA score stayed the same or increased, or who died during the 7-day follow-up

**Fig. 2:**
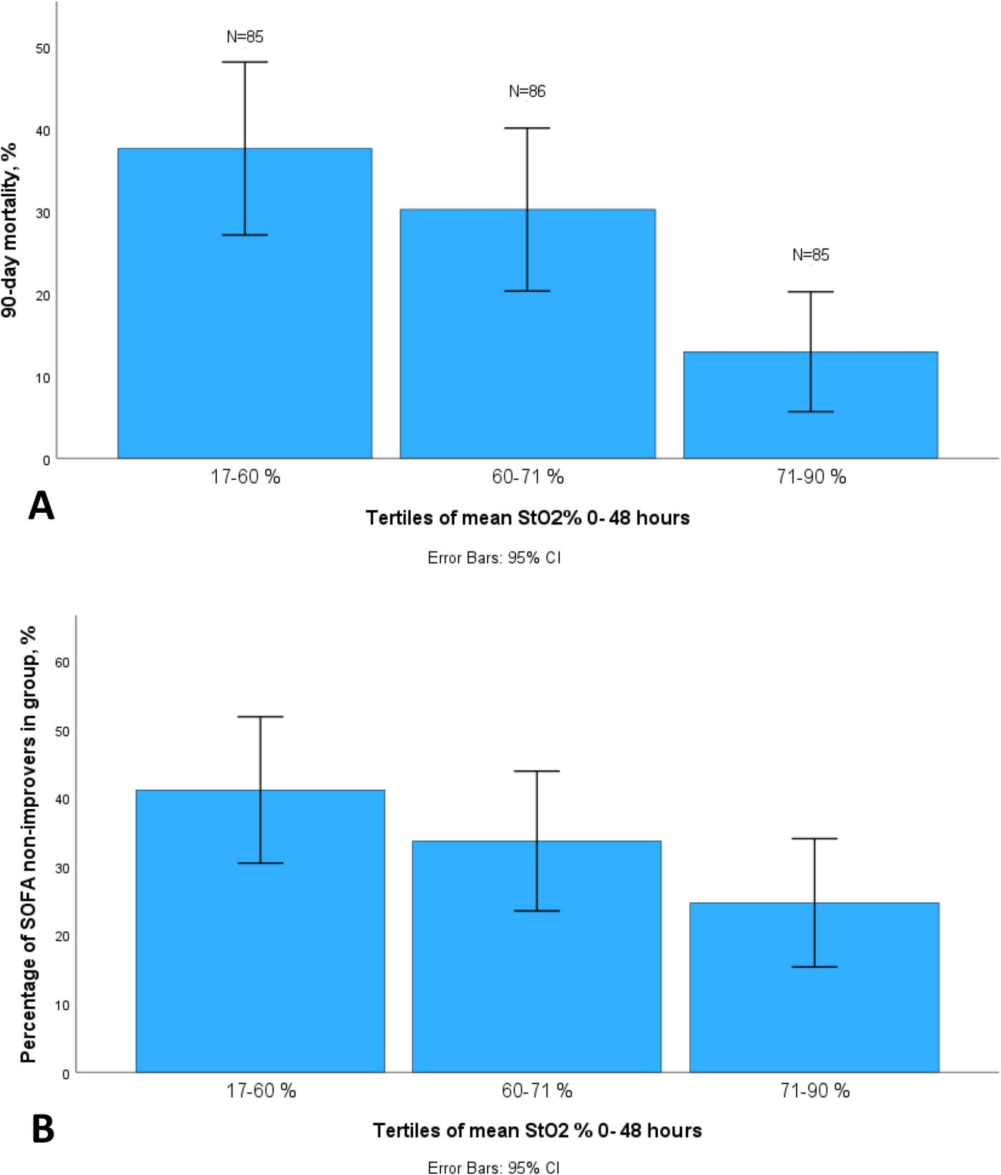
Tertiles of mean 48-h StO_2_ and organ dysfunction (**a**) or 90-day mortality (**b**). SOFA-improvers were patients, whose SOFA score decreased during the 7 day follow-up, and SOFA-non-improvers patients, whose SOFA score stayed the same or increased, or who died during the 7-day follow-up

Figure 1a and b illustrate the evolution of mean StO_2_ over time, and Figs. S6a and b (ESM) exluding the last six hours of the StO_2_ registration in patients who died during the registration. Figure 2a and b show the tertiles of 48-h mean StO_2_ according to development of organ dysfunction and 90-day mortality. In logistic regression models 48-h mean StO_2_ was associated with 90-day mortality (odds ratio [OR] 0.97, CI 95% 0.94–1.00, *p* = 0.047) but not with change in SOFA score (Table 3). Mottling score also, was associated with 90-d mortality (OR 1.84, CI 95% 1.10–3.08) but not with SOFA change (Table 3). Tables S6a and b (ESM) show the additional properties of diagnostic accuracy. When the last six hours of the StO_2_ registration for those that died during the registration was excluded, 48-h mean StO_2_ was no longer associated with 90-day mortality in the logistic regression analyses (OR 0.97, CI 95% 0.94–1.00, *p* = 0.062) (Table S7 ESM).To explore the influence of short term changes in clinical parameters, we calculated delta variables as a difference from first to last observation hour within the first 48 h (Table S8, ESM). For StO_2_ we also calculated deltas for the first 12 and 24 h (Figs. S7a, b, c and d ESM). We also performed sensitivity analyses for the sub-group of patients with sepsis (Tables S9, S10 and S11, ESM), and the results did not change for StO_2_ or mottling score.

**Table 3:** Results of logistic regression models for organ dysfunction and 90-day mortality

	Organ dysfunction		90-day mortality	
Variables in the model	Odds ratio	*p*	Odds ratio	*p*
	(95% Confidence interval)		(95% Confidence interval)	
Model 1				
Capillary refill time^a^	–	ns	–	ns
Chronic kidney disease	2.37 (1.00–5.63)	0.05	2.84 (1.06–7.65)	0.039
Lactate^a^	1.27 (1.09–1.47)	0.002	1.32 (1.11–1.58)	0.002
Mean arterial pressure^a^	0.91 (0.87–0.97)	0.001	0.92 (0.86–0.98)	0.011
Norepinephrine dose^a^	–	ns	–	ns
Peripheral StO_2_^a^	–	ns	0.97 (0.94–1.00)	0.047
SAPS II score^b^	–	ns	1.04 (1.01–1.07)	0.005
Smoker (ever)	2.52 (1.34–4.72)	0.004	2.36 (1.10–5.07)	0.028
Model 2				
Capillary refill time^a^	–	ns	–	ns
Chronic kidney disease	–	ns	3.25 (1.21–8.75)	0.02
Lactate^a^	1.27 (1.10–1.48)	0.002	1.28 (1.08–1.52)	0.004
Mean arterial pressure^a^	0.91 (0.87–0.96)	< 0.001	0.92 (0.86–0.98)	0.012
Mottling score^a^	–	ns	1.84 (1.10–3.08)	0.02
Norepinephrine dose^a^	–	ns	–	ns
SAPS II score^b^	–	ns	1.04 (1.01–1.07)	0.004
Smoker (ever)	2.47 (1.32–4.62)	0.005	2.39 (1.10–5.17)	0.028

Binary logistic regression models (forward stepwise, conditional). Model 1 contains peripheral 48-h mean StO_2_ and Model 2 contains 48-h mean mottling score instead

Organ dysfunction was defined as unchanged or worsening SOFA score or death within 7 days in intensive care unit. Chronic kidney disease (moderate or severe) was defined as glomerular filtration rate < 60 ml/minute/1.73 m^2^ or chronic dialysis at least one week prior to ICU admission

*SAPS II* Simplified acute physiology score; *MAP* Mean arterial pressure; *StO*_*2*_ Tissue Oxygen Saturation; *ns* Not significant

^a^48-h mean

^b^0–24 h

### Feasibility of NIRS monitoring

The median duration of NIRS monitoring was 47.6 (IQR 40.5; 48.0) hours and the proportion of missing data was 2.2% (IQR 0.2; 8.8). Feasibility data were missing in 16 (6.3%) patients. The median monitoring feasibility score was 0.3/5.0 (IQR 0.0; 0.8). The most frequent problems were detachment of the cords and/or probes in 34 (14.2%, n = 240) patients. Patient related problems were reported in 6 (2.5%, n = 240) patients (ESM Table S12).

## Discussion

In this prospective, observational study of 256 critically ill adult patients with circulatory shock with continuous peripheral NIRS StO_2_ monitoring, 48-h mean StO_2_ was lower in patients with persisting or worsening organ dysfunction, and in patients who died within 90 days. In a multivariable model, however, mean 48-h StO_2_ was independently associated with 90-day mortality, but not with evolution of organ dysfunction. The association with mortality was however not significant when the last six hours of StO_2_ registration of those patients who died during the registration was excluded from the analyses. NIRS monitoring in the ICU was well tolerated and feasible.

Our findings are in line with earlier studies exploring peripheral tissue oxygenation and adverse outcome in patients with shock. Low thenar StO_2_ during the first 3 h, and at 6 h after the arrival in the emergency department predicted organ dysfunction and mortality in three studies on level 1 trauma center patients with circulatory shock [21–23]. In two studies on mixed shock populations low thenar StO_2_, that persisted despite resuscitation, was associated with more severe organ failure [24], but not with mortality [25]. In previous studies and in a systematic review and meta-analysis of sepsis and septic shock patients, peripheral StO_2_ predicted these adverse outcomes, however, the association was more consistent when StO_2_ was measured at the knee or at the brachial muscle, than when measured at the thenar eminence [4, 10, 26–28].

In a previous study on patients with septic shock and high mortality, the AUROC values of knee StO_2_ for predicting mortality were high, over 0.85 and similar to that of mottling of the skin of the knee region [4]. The AUROC values of the present study were lower than in previous studies, but also predictive of both organ dysfunction and mortality. This difference in predictive capacity between the present study and previous ones could at least partly be explained by the higher mortality rates of up to 57.9% in earlier studies [4, 26, 27]. Accordingly, in two groups of septic and septic shock patients with lower mortality rates, 5% and 38% respectively, Shapiro et al. reported AUROC values similar to those of the present study [29]. Moreover, the heterogeneity of the patient population of the present study might have affected the results, as the mechanisms behind tissue perfusion impairment vary in different types of shock [30]. To better account for the variability in StO_2_ during the clinical course of shock, and express the burden of tissue hypoperfusion, we calculated the AUT and time below the predefined StO_2_ thresholds, 40%, 50% and 60% and above StO_2_ 80%. AUTs and time below the studied StO_2_ thresholds were associated with mortality but not with evolution of organ dysfunction, and somewhat surprisingly, they did not improve outcome prediction (Tables 2, S2a, b, S3a and b [ESM]).

In general, all the measured hemodynamic and microcirculatory parameters in the present study seemed to be associated with 90-day mortality rather than organ dysfunction. One possible explanation could be that in some patients of the present study, organ dysfunction may have taken longer than seven days to resolve. Changes in SOFA score after day 7 were not recorded in the present study but may have had an impact on mortality at 90 days. Also, although all changes in different SOFA sub-scores are associated with mortality, the weighs of the different sub scores may not be equal for mortality prediction, which may have had an impact on the results of the present study. [31, 32] In our study, however, the highest SOFA scores were mostly measured during the first 24 h of the study, meaning the patients arrived in the ICU with organ dysfunction already present. In addition, we found that decreasing or stagnant StO_2_ values over the first 12 or 24 h of the shock were associated with adverse outcomes. This supports the use of StO_2_ in predicting the clinical course of organ dysfunction, or death, rather than organ dysfunction as such.

In the multivariable analyses, knee 48-h mean StO_2_ was independently associated with 90-day mortality, but it was not independently associated with evolution of organ dysfunction. When mottling score was included in the analyses instead of 48-h mean StO_2_, we found that mottling score was independently associated with mortality. The similarity and interchangeability of knee region StO_2_ and mottling score is physiologically reasonable, since the two variables are correlated, and StO_2_ measured at the suprapatellar region of the knee could be regarded as a numerical expression of mottling [4]. The association of StO_2_ with mortality was, however, no longer significant when the last six hours of the StO_2_ registration of patients who died during the 48-h StO_2_ registration period was excluded from the multivariable regression analyses. A high proportion (83%) of these early deaths was preceded by a decision to withdraw life sustaining therapy, which may be considered a confounding factor. Exclusion of the last hours of registration in these very ill patients may, however, also have lost the signals of rapid deterioration prior to treatment limitation, which may have had a negative effect on the predictive capacity of StO_2_.

Regional NIRS-derived perfusion disturbances of the skin show a relation to other disturbances of peripheral perfusion [33] and to more global markers of hypoperfusion, such as elevated lactate levels [4]. Previous studies have demonstrated the association of different microcirculatory or tissue perfusion parameters, such as mottling score, CRT, and blood lactate level, with patient outcomes in circulatory shock [3, 34–36]. In our study, blood lactate but not CRT was independently associated with both mortality and SOFA change in multivariable analyses, whereas mottling score was only associated with mortality. However, unlike those parameters, StO_2_ monitoring provides continuous, non-invasive and objective, numerical data, that does not require repeated clinical examination, invasive measurements, or blood sampling. Furthermore, in our study, StO_2_ was associated with mortality even in patients without mottling. This could be explained by the categorical nature of the mottling score or, to some extent, subjective variation in assessment of the score. However, it could also mean that StO_2_ can detect subtle changes in tissue perfusion better than assessment of the mottling score. Nevertheless, mottling score, which is also non-invasive, might predict mortality equally well or even better than StO_2_.

Optimizing tissue perfusion is indisputably the goal of hemodynamic resuscitation in circulatory shock [12]. The optimal methods for monitoring tissue perfusion, however, are not agreed on. Moreover, no monitoring modality can produce better results than the treatment it guides [37], and the optimal targets and resuscitation algorithms for improving tissue perfusion in various clinical scenarios and heterogenous patient populations with shock are still largely unknown [38]. In our study, patients with higher mean StO_2_ and higher area over StO_2_ 80%, had better outcomes. Previously only few studies have explored different methods of modifying peripheral StO_2_ in shock. For example, both norepinephrine and nitroglycerin administration, but not red blood cell transfusion have been shown to improve peripheral StO_2_ in observational studies [39–41]. Clinical trials on StO_2_ guided hemodynamic treatment in shock are even more scarce, but in a recent randomized controlled trial targeting StO_2_ over 80% using a hemodynamic treatment algorithm including central venous pressure and MAP targets, red blood cell transfusions and dobutamine use, did not seem to affect outcomes in patients with severe sepsis and septic shock [14]. Furthermore, StO_2_ levels are known to vary over different anatomical monitoring sites [4], and significant inter-individual variation might support the use of individualized rather than fixed StO_2_ targets. [42] Also, it is worth noting, that results acquired with different NIRS-monitors and probes are not directly comparable [43].

Finally, earlier findings together with the findings of the present study indicate that StO_2_ can be used as a quantitative means of assessing tissue perfusion, and potentially also serve as an indirect means of assessing end-organ hypoperfusion in general. The next phase would be to explore different means to modify peripheral StO_2_ and eventually, perhaps, patient outcomes.

### Strengths and limitations of the study

With 256 patients, our study is to date, the largest prospective observational study on continuous peripheral NIRS monitoring in circulatory shock. Furthermore, including all types of shock patients supports the generalizability of the results. Our study demonstrates the association of peripheral StO_2_ and mortality in a large cohort of patients with early phase of shock, regardless of the underlying mechanisms of shock. In addition, the personnel were blinded regarding the NIRS monitoring. Also, one of the study’s strengths is the completeness of the data. The median duration of NIRS monitoring was over 46 h and the proportion of missing data was small. Moreover, the proportion of patients with missing outcome data on organ dysfunction and mortality was negligible.

There are also several limitations of the study. Firstly, changes in SOFA score during the first week may not always detect development or alleviation of organ dysfunction, and changes occurring after the first seven days were not measured or analyzed in the present study. Secondly, due to the COVID-19 pandemic there were several interruptions in the recruitment process. Thirdly, night-time recruitment was carried out by the ICU clinical personnel, therefore the included patients were not always consecutive, and the patient sample may be regarded as a convenience sample. In addition, the decision to exclude patients likely to be transferred to the ward within 24 h, may have led to inclusion of patients with more severe shock. These limitations may have created potential bias. Moreover, for statistical analyses, we decided to dichotomize the primary outcome as opposed to the original sample size calculations and we did not adjust for multiple comparisons. Also, because the study was planned and commenced before the COVID-19 pandemic, we did not record the number of patients with COVID-19 as a diagnosis. Furthermore, we chose not perform a vascular occlusion test to obtain dynamic NIRS parameters, due to the lack of established measurement procedures in the lower limb in the critically ill population. Finally, a total of 29 (42%) of the 69 deaths occurred within the 48-h StO_2_ monitoring period and a majority (83%) of these early deaths was preceded by a decision to withdraw life sustaining therapy.

## Conclusions

48-h mean peripheral StO_2_ was not associated with persisting or worsening organ dysfunction. StO_2_ was, however, lower in patients who died within 90 days and more importantly, it was independently associated with 90-day mortality in circulatory shock. The adjusted association with mortality was not significant when the last six hours of StO_2_ registration of the patients who died during the 48-h StO_2_ registration was excluded from the analyses. Continuous peripheral StO_2_ monitoring using NIRS in the ICU was feasible. Continuous peripheral NIRS monitoring may provide a non-invasive method for assessment of tissue perfusion.

## Supplementary Information

Additional file1 (PDF 1002 kb)

Additional file2 (JPG 131 kb)

## Data Availability

The datasets generated and analysed during the current study are available from the corresponding author on reasonable request.

## Abbreviations

AKI: Acute kidney injury
APACHE: Acute Physiology and Chronic Health Evaluation
AOT: Area over StO_2_ threshold (Area under curve above StO_2_ threshold, see Fig. S1 ESM)
AUT: Area under StO_2_ threshold (Area under curve below StO_2_ threshold, see Fig. S1 ESM)
AUROC: Area under the receiver operating characteristics curve
BE: Base excess
CI: Confidence interval
CRRT: Continuous renal replacement therapy
CRT: Capillary refill time
ECG: Electrocardiography
ESM: Electronic supplementary material
ICU: Intensive care unit
IHD: Intermittent hemodialysis
IQR: Interquartile range
KDIGO: Kidney Disease Improving Global Outcomes
MAP: Mean arterial pressure
NIRS: Near-infrared spectroscopy
OR: Odds ratio
RRT: Renal replacement therapy
SAPS II: Simplified Acute Physiology Score II
SOFA: Sequential Organ Failure Assessment
SOP: Standard operating procedures
StO_2_: Tissue oxygen saturation
STROBE: Strengthening the Reporting of Observational Studies in Epidemiology
