# Supplementary material for: Clinical outcomes and peripheral tissue oxygen saturation monitoring of the knee region by near-infrared spectroscopy in circulatory shock: a prospective observational cohort study
Source: Crit Care. 2025 Mar 19;29:125. doi: 10.1186/s13054-025-05363-1 (PMC11924835; doi:10.1186/s13054-025-05363-1)
Supplement: Supplementary file 1 — Additional file1 (PDF 1002 kb) [file 13054_2025_5363_MOESM1_ESM.pdf]

Supplementary material

Clinical outcomes and peripheral tissue oxygen saturation monitoring of the knee region by near-infrared spectroscopy in circulatory shock: a prospective observational cohort study

Critical Care

Elina Varis[1], Maria Heliste[1], Johanna Hästbacka[2], Suvi T Vaara[1], Markus B Skrifvars[1], Ville Pettilä[1], Mitja Lääperi[3], Anne Kuitunen[2], Annukka Vahtera[2], Erika Wilkman[1]

Author Information:

Affiliations:

1. Helsinki University Hospital (Perioperative and Intensive Care) and University of Helsinki, Helsinki, Finland
2. Tampere University Hospital, Wellbeing Services County of Pirkanmaa, and Tampere University, Faculty of Medicine and Health Technology, Tampere, Finland
3. Lääperi Statistical Consulting, Espoo, Finland

Corresponding author:

Elina Varis, elina.varis@hus.fi

Index page

Fig. S1 Visual presentation of AUT and AOT calculations ……………………………… 4

Fig. S2 Study flowchart……………………………………………………………………… 5

Table S1 Additional patient characteristics……………………………………………….. 6

Fig. S3a Organ dysfunction and signs of tissue hypoperfusion……………………………..8

Fig. S3b 90-day mortality and signs of tissue hypoperfusion……………………………….8

Fig. S4 48-hour mean StO2 and SOFA change…………………………………………… 9

Table S2a Additional results of univariable analyses for organ dysfunction………………….. 10

Table S2b Additional results of univariable analyses for 90-day mortality…………………… 12

Table S3a Results of AUROC analyses for organ dysfunction………………………………… 14

Table S3b Results of AUROC analyses for 90-day mortality…………………………….... 15

Fig. S5a ROC curves at predicting organ failure…………………………………………. 16

Fig. S5b ROC curves at predicting 90-day mortality……………………………………….. 17

Table S4 Early versus late deaths…………………………………………………………….. 18

Table S5 Results of univariable analyses excluding the last six hours' data pre-death……….. 19

Fig. S6a Mean StO_2 and organ dysfunction excluding the last six hours' data pre-death……….20

Fig S6b Mean StO_2 and 90-day mortality excluding the last six hours' data pre-death ………. 20

Table S6a Additional diagnostic properties for predicting organ dysfunction…………………...21

Table S6b Additional diagnostic properties for predicting death at 90 days……………………22

Table S7 Results of logistic regression models excluding the last six hours' data pre-death.... 23

Table S8 Results of univariable and adjusted models of delta variables………………………..24

Fig. S7a Tertiles of delta StO2 over the first 12 hours and organ dysfunction…………………. 25

Fig. S7b Tertiles of delta StO2 over the first 24 hours and organ dysfunction…………………. 25

Fig. S7c Tertiles of delta StO2 over the first 12 hours and 90-day mortality……………………26

Fig. S7d Tertiles of delta StO2 over the first 24 hours and 90-day mortality……………………26

Table S9 Patient characteristics of sepsis sub-group……………………………………………. 27

Table S10 Results of univariable analyses for sepsis sub-group………………………………... 28

Table S11 Results of logistic regression models for sepsis sub-group………………………… 30

Table S12 Reported problems in NIRS-monitoring…………………………………………... 31

References……………………………………………………………………………………..32

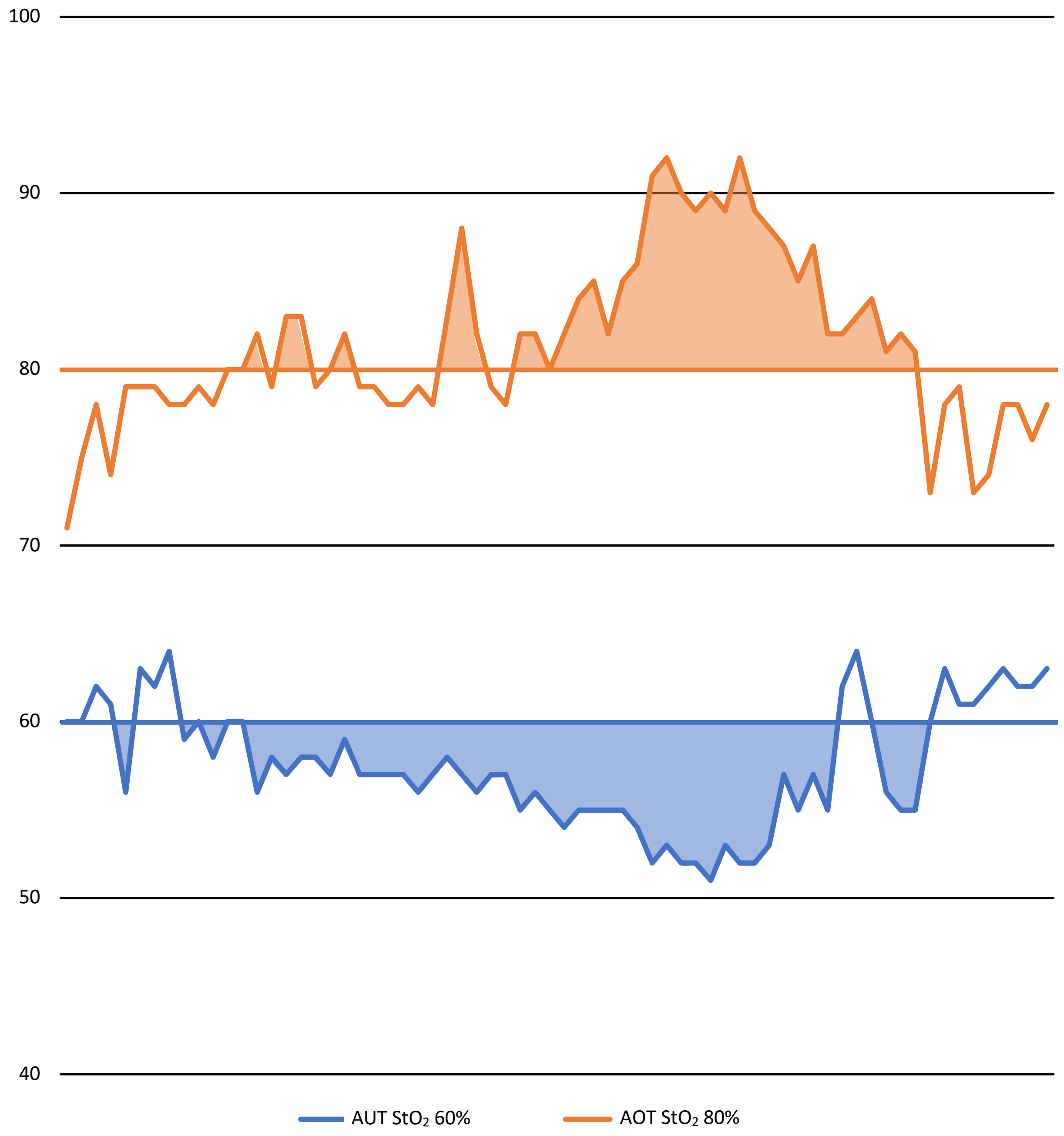

Fig. S1 Visual presentation of calculations of area under the curve, below and above specific StO_2 thresholds. The areas under threshold (AUT; StO_2 [%] x time [minutes]) below the three prespecified StO_2 thresholds (60%, 50% and 40%) were calculated as follows: All StO_2 values exceeding the threshold value were excluded from the data. The threshold value was then subtracted from the patient StO_2 values and an area under the curve was calculated for the inverse of the differences. Post hoc, we also decided to calculate area over threshold (AOT) above StO_2 80% in similar manner.

.

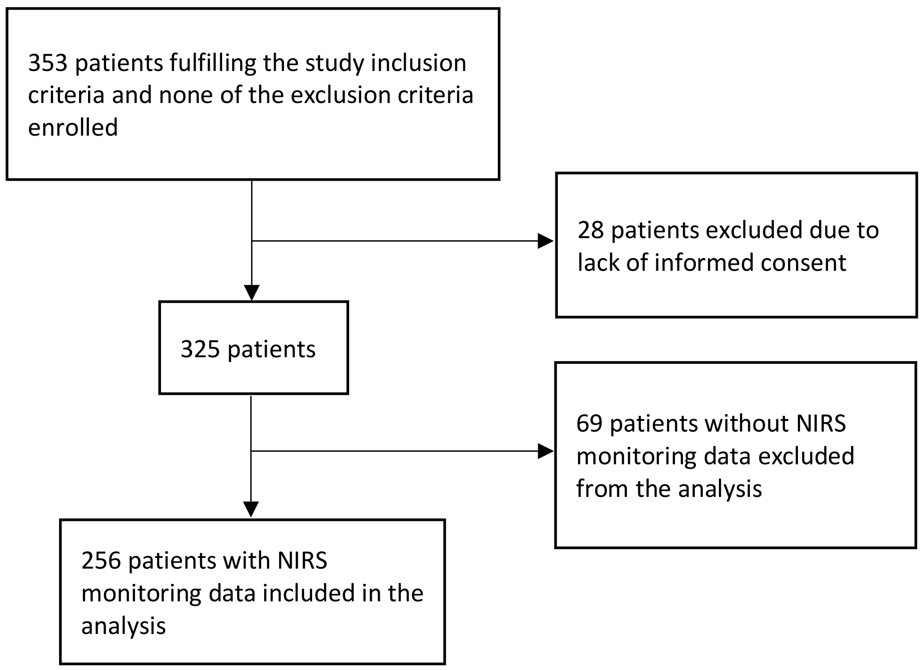

Fig. S2 Study Flowchart. During the enrollment period from from April 2019 to May 2023 the enrollment was paused due to the COVID-19 pandemic from 30 March 2020 to 31 May 2020 and from 28 February 2021 to 19 April 2021

Table S1 Additional patient characteristics

			Organ dysfunction			90-day mortality		
	Missing n	All patients n=256	SOFA-improvers n=171	SOFA-non-improvers n=85	p	90-d survivors n=187	90-d non-survivors n=69	p
**Number of signs of tissue hypoperfusion at enrollment**	0	2 (2; 3)	2 (1; 3)	3 (2; 4)	0.194	2 (1; 3)	3 (2; 4)	0.001
0		18 (7.0)	14 (8.2)	4 (4.7)	0.766	16 (8.6)	2 (2.9)	0.051
1		43 (16.8)	30 (17.5)	13 (15.3)		34 (18.2)	9 (13.0)	
2		79 (30.9)	54 (31.6)	25 (29.4)		63 (33.7)	16 (23.2)	
3		54 (21.1)	34 (19.9)	20 (23.5)		37 (19.8)	17 (24.6)	
4		46 (18.0)	29 (17.0)	17 (20.0)		28 (15.0)	18 (26.1)	
5		13 (5.1)	9 (5.3)	4 (4.7)		8 (4.3)	5 (7.2)	
6		3 (1.2)	1 (0.6)	2 (2.4)		1 (0.5)	2 (2.9)	
**Body Mass Index (kg/m2)**	3	26.6 (23.0-31.2)	26.5 (23.1-31.1)	26.7 (22.9-31.9)	0.935	26.9 (23.0-31.2)	26.1 (22.8-31.2)	0.603
**Admission type surgical**	0	129 (50.4)	85 (49.7)	44 (51.8)	0.792	92 (49.2)	37 (53.6)	0.574
**Vasoactive medication**								
Norepinephrine	0	256 (100.0)	171 (100.0)	85 (100.0)	-	187 (100.0)	69 (100.0)	-
Epinephrine	3	7 (2.8)	4 (2.4)	3 (3.6)	0.688	5 (2.7)	2 (2.9)	1.000
Levosimendan	3	18 (7.1)	13 (7.7)	5 (6.0)	0.796	13 (7.0)	5 (7.3)	1.000
Vasopressin	3	3 (1.2)	2 (1.2)	1 (1.2)	1.000	2 (1.1)	1 (1.5)	1.000
Dobutamine	3	8 (3.2)	3 (1.8)	5 (6.0)	0.121	3 (1.6)	5 (7.4)	0.035
Dopamine	3	10 (4.0)	8 (4.7)	2 (2.4)	0.504	9 (4.9)	1 (1.5)	0.296
**AKI and RRT**								
AKI	0	167 (65.2)	103 (60.2)	64 (75.3)	0.018	106 (56.7)	61 (88.4)	<0.001
AKI duration (days) (in AKI patients)	0	3.0 (2.0-5.0)	3.0 (2.0-6.0)	2.0 (1.0-4.0)	0.007	3.0 (2.0-5.3)	2.0 (2.0-5.0)	0.281
AKI max stage (in AKI patients)	0	3 (2.0-3.0)	3.0 (2.0-3.0)	3.0 (2.0-3.0)	0.047	3.0 (1.0-3.0)	3.0 (2.0-3.0)	0.045
RRT (CRRT or IHD or both)	0	80 (31.3)	39 (22.8)	41 (48.2)	<0.001	45 (24.1)	35 (50.7)	<0.001
CRRT	1	68 (26.6)	29 (17.0)	39 (45.8)	<0.001	35 (18.7)	33 (47.8)	<0.001
IHD	1	20 (7.8)	17 (9.9)	3 (3.5)	0.086	17 (9.1)	3 (4.3)	0.296
RRT duration (days)	0	2.0 (2.0-5.0)	4.0 (2.0-6.0)	2.0 (2.0-4.5)	0.073	4.0 (2.0-5.0)	2.0 (2.0-5.0)	0.183
SOFA change from day 1 to day 7 or ICU discharge	2	-2 (-5; 0)	-4 (-6; -2)	0 (0; 1)	0.000	-3 (-5; -1)	0 (-2; 1)	<0.001

Daily SOFA score								
day 1 (n=256)	2	10.0 (8.0-13.0)	9.0 (8.0-12.0)	10.0 (7.0-13.0)	0.126	9.0 (7.0-11.0)	13.0 (9.0-15.0)	<0.001
day 2 (n=240)	16	9.0 (7.0-12.0)	8.0 (6.0-11.0)	11.0 (9.0-15.0)	<0.001	8.0 (6.0-11.0)	12.0 (9.0-15.0)	<0.001
day 3 (n=185)	13	8.0 (6.0-11.0)	8.0 (5.0-10.5)	11.0 (9.0-15.0)	<0.001	8.0 (6.0-11.0)	11.0 (9.0-14.0)	<0.001
day 4 (n=153)	16	8.0 (5.0-11.0)	7.0 (5.0-10.0)	12.5 (9.8-16.0)	<0.001	7.0 (4.0-10.0)	11.0 (9.0-14.0)	<0.001
day 5 (n=132)	13	8.0 (5.0-11.0)	7.0 (4.0-10.0)	13.0 (9.0-15.0)	<0.001	6.0 (4.0-10.0)	10.5 (9.0-13.8)	<0.001
day 6 (n=106)	16	8.0 (5.0-11.0)	7.0 (4.0-10.5)	12.0 (8.5-16.0)	<0.001	7.0 (4.0-11.0)	11.0 (7.8-14.5)	<0.001
day 7 (n=84)	10	7.0 (5.0-11.0)	7.0 (5.0-9.5)	12.0 (8.0-15.0)	<0.001	7.0 (5.0-10.0)	9.5 (7.0-14.0)	0.006

Continuous variables are summarized as medians with interquartile ranges (IQR), categorical variables as counts and percentages.
Organ dysfunction was defined as unchanged or worsening SOFA score or death within 7 days in intensive care unit.
Abbreviations: SOFA-improvers, patients whose SOFA score decreased during the 7 day follow-up; SOFA-non-improvers, patients whose SOFA score stayed the same or increased, or who died during the 7-day follow-up; SOFA, Sequential Organ Failure Assessment; ICU, intensive care unit; AKI, Acute Kidney Injury, defined by Kidney Disease Improving Global Outcomes (KDIGO) Clinical Practice Guidelines [1]; RRT, Renal Replacement Therapy; CRRT, Continuous Renal Replacement Therapy; IHD, Intermittent Hemodialysis.

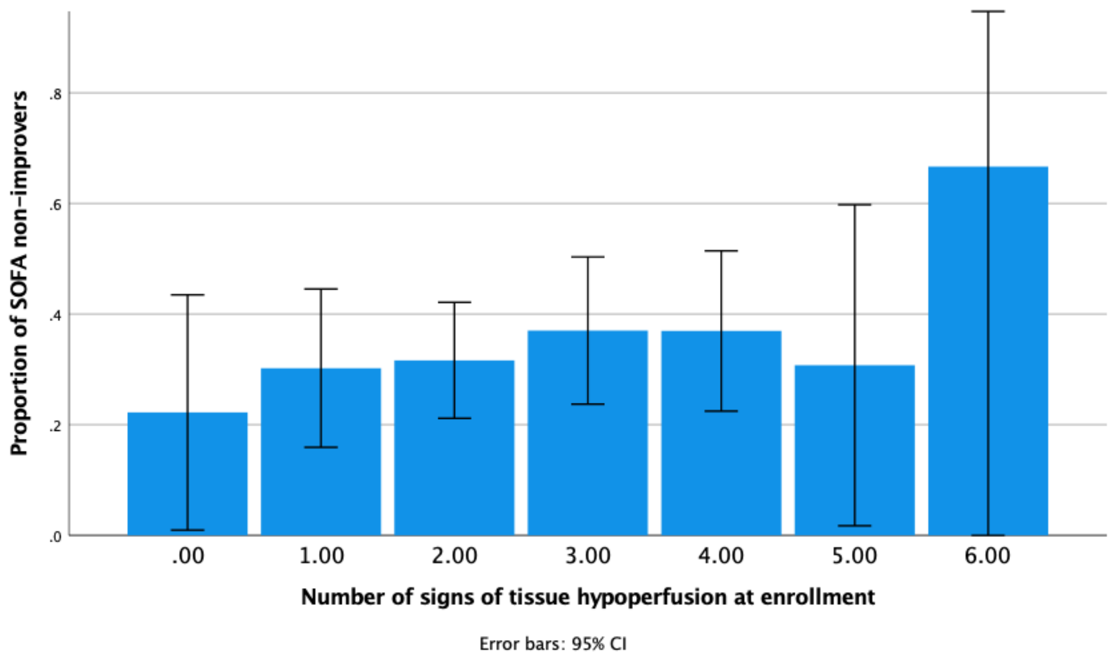

Fig. S3a Proportion of SOFA non-improvers and number of signs of tissue hypoperfusion at study enrollment. Abbreviations: SOFA, Sequential Organ Failure Assessment; SOFA-non-improvers, patients whose SOFA score stayed the same or increased, or who died during the 7-day follow-up.

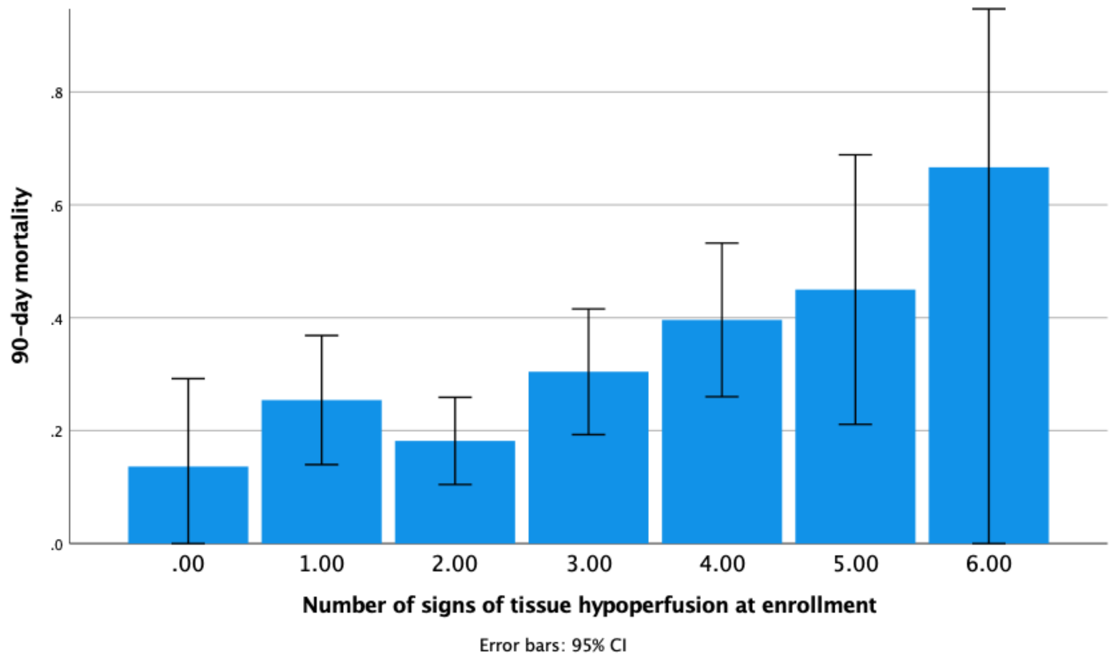

Fig. S3b 90-day mortality and number of signs of tissue hypoperfusion at study enrollment.

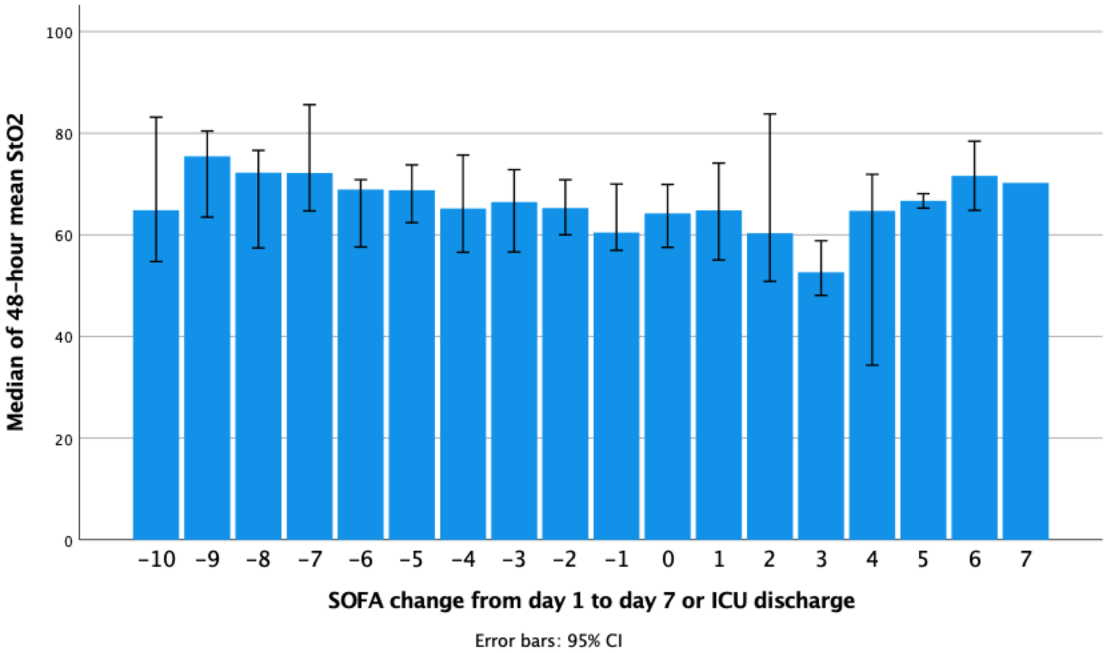

Fig. S4 Median of 48-hour mean StO_2 and SOFA change from day 1 to day 7 or ICU discharge. Abbreviations: StO_2, tissue oxygen saturation; SOFA, Sequential Organ Failure Assessment; ICU, intensive care unit.

Table S2a Results of univariable analyses for organ dysfunction, additional time-intervals and parameters.

		Organ dysfunction		
	Missing n	SOFA-improvers n=171	SOFA-non-improvers n=85	p
**Mean arterial pressure (mmHg)**				
**0-6 h**	3	71.1 (66.6-76.3)	70.3 (64.1-73.2)	0.072
**0-24 h**	3	72.9 (68.6-76.5)	69.3 (65.0-73.6)	0.073
**Heart rate (bpm)**				
**0-6 h**	3	93.5 (79.4-107.9)	95.7 (79.7-110.8)	0.629
**0-24 h**	3	91.2 (78.7-103.2)	94.3 (80.7-109.2)	0.511
**Norepinephrine dose (μg/kg/min)[a]**				
**0-6 h**	4	0.18 (0.11-0.35)	0.25 (0.12-0.49)	0.032
**0-24 h**	4	0.14 (0.08-0.31)	0.27 (0.08-0.55)	0.004
**Arterial lactate (mmol/l)**				
**0-6 h**	13	2.4 (1.5-4.3)	3.3 (1.7-5.8)	0.026
**0-24 h**	12	1.9 (1.3-3.3)	3.3 (1.5-5.5)	<0.001
**Capillary refill time (s)**				
**0-6 h**	2	5.4 (3.4-8.9)	6.3 (3.2-9.8)	0.612
**0-24 h**	0	5.1 (3.2-8.2)	6.8 (3.2-12.1)	0.040
**Mottling score**				
**0-6 h**	1	0.0 (0.0-1.0)	0.0 (0.0-1.0)	0.931
**0-24 h**	0	0.0 (0.0-0.4)	0.1 (0.0-0.9)	0.050
**Peripheral temperature (°C)**				
**0-6 h**	6	31.9 (30.4-33.4)	31.9 (29.9-33.2)	0.404
**0-24 h**	1	32.8 (31.3-33.8)	32.3 (30.7-33.6)	0.141
**Central to peripheral temperature gradient (°C)**				
**0-6 h**	20	4.7 (3.2-6.6)	4.4 (2.3-6.4)	0.328
**0-24 h**	7	4.4 (2.7-5.9)	3.9 (2.4-5.6)	0.440
**Mean Peripheral StO_2 (%)**				
**0-6 h**	4	64.6 (54.2-73.1)	63.6 (55.0-71.8)	0.711
**0-24 h**	2	66.9 (55.9-73.6)	62.8 (53.5-70.8)	0.105
**AUT below StO_2 thresholds (%min)**				
**60 %**				
**0-6 h**	0	86.3 (0.0-2039.3)	192 (0.0-1607.8)	0.925
**0-24 h**	0	661.5 (0.0-6635.3)	850.8 (0.4-8071.1)	0.536
**50 %**				
**0-6 h**	0	0.0 (0.0-233.8)	0.0 (0.0-174.9)	0.855
**0-24 h**	0	4.5 (0.0-809.0)	0.0 (0.0 t0 1870.3)	0.912
**40 %**				
**0-6 h**	0	0.0 (0.0-0.0)	0.0 (0.0-0.0)	0.787
**0-24 h**	0	0.0 (0.0-25.3)	0.0 (0.0-144.0)	0.357
**AOT above StO_2 80 % (%min)**				
**0-6 h**	0	0.0 (0.0-0.0)	0.0 (0.0-0.0)	0.332
**0-24 h**	0	0.0 (0.0-418.5)	0.0 (0.0-26.0)	0.039
**Time below StO_2 thresholds (min)**				
**60 %**				
**0-6 h**	4	37.8 (0.0-268.1)	46.0 (0.0-182.4)	0.744
**0-24 h**	2	118.5 (0.0-885.1)	259.8 (0.63-811.6)	0.789
**50 %**				
**0-6 h**	4	0.0 (0.0-51.4)	0.0 (0.0-52.5)	0.805
**0-24 h**	2	0.5 (0.0-193.6)	0.0 (0.0-287.9)	0.927
**40 %**				
**0-6 h**	4	0.0 (0.0-0.0)	0.0 (0.0-0.0)	0.556
**0-24 h**	2	0.0 (0.0-2.9)	0.0 (0.0-14.5)	0.660
**Time above StO_2 80 % (min)**				

**0-6 h**	4	0.0 (0.0-0.0)	0.0 (0.0-0.0)	0.363
**0-24 h**	2	0.0 (0.0-95.3)	0.0 (0.0-11.5)	0.046
**AUT below StO_2 thresholds divided by the length of follow-up (%)**				
**<u>60 %</u>**				
**0-6 h**	0	0.34 (0.00-6.08)	0.57 (0.00-4.95)	0.976
**0-24 h**	0	0.52 (0.00-5.01)	0.65 (0.00-6.94)	0.387
**0-48 h**	1	0.77 (0.02-4.67)	1.07 (0.03-8.79)	0.158
**<u>50 %</u>**				
**0-6 h**	0	0.00 (0.00-0.68)	0.00 (0.00-0.95)	0.892
**0-24 h**	0	0.00 (0.00-0.64)	0.00 (0.00-1.80)	0.806
**0-48 h**	1	0.02 (0.00-0.81)	0.06 (0.00-2.36)	0.407
**<u>40 %</u>**				
**0-6 h**	0	0.00 (0.00-0.00)	0.00 (0.00-0.00)	0.795
**0-24 h**	0	0.00 (0.00-0.02)	0.00 (0.00-0.13)	0.322
**0-48 h**	1	0.00 (0.00-0.10)	0.00 (0.00-0.36)	0.153
**AOT above StO_2 80 % divided by the length of follow-up (%)**				
**0-6 h**	0	0.00 (0.00-0.00)	0.00 (0.00-0.00)	0.324
**0-24 h**	0	0.00 (0.00-0.38)	0.00 (0.00-0.02)	0.036
**0-48 h**	1	0.02 (000-0.70)	0.00 (0.00-0.04)	<0.001
**Time below StO_2 thresholds divided by the length of follow-up (%)**				
**<u>60 %</u>**				
**0-6 h**	4	12.5 (0.0-91.9)	15.9 (0.0-85.0)	0.927
**0-24 h**	2	9.9 (0.0-68.5)	19.0 (0.1-81.8)	0.391
**0-48 h**	1	8.7 (0.3-58.6)	21.1 (1.3-80.9)	0.114
**<u>50 %</u>**				
**0-6 h**	4	0.0 (0.0-16.6)	0.0 (0.0-20.2)	0.823
**0-24 h**	2	0.0 (0.0-14.5)	0.0 (0.0-26.8)	0.905
**0-48 h**	1	0.2 (0.0-12.5)	0.5 (0.0-33.6)	0.431
**<u>40 %</u>**				
**0-6 h**	4	0.0 (0.0-0.0)	0.0 (0.0-0.0)	0.569
**0-24 h**	2	0.0 (0.0-0.3)	0.0 (0.0-1.3)	0.598
**0-48 h**	1	0.0 (0.0-0.8)	0.0 (0.0-5.1)	0.289
**Time above StO_2 80 % divided by the length of follow-up (%)**				
**0-6 h**	4	0.0 (0.0-0.0)	0.0 (0.0-0.0)	0.341
**0-24 h**	2	0.0 (0.0-7.7)	0.0 (0.0-1.0)	0.046
**0-48 h**	1	0.6 (0.0-18.7)	0.0 (0.0-2.3)	0.001

All continuous parameters are summarized as medians and interquartile ranges (IQR).
Organ dysfunction was defined as unchanged or worsening SOFA score or death within 7 days in intensive care unit.
Abbreviations: StO_2, tissue oxygen saturation; SOFA, Sequential Organ Failure Assessment; h, hour; SOFA-improvers, patients whose SOFA score decreased during the 7 day follow-up; SOFA-non-improvers, patients whose SOFA score stayed the same or increased, or who died during the 7-day follow-up; mmHg, millimeters of mercury; bpm, beats per minute; µg/kg/min, micrograms per kilogram per minute; mmol/l, millimoles per liter; s, seconds; °C, degrees of Celsius; AUT, area under StO_2 threshold; AOT, area over StO_2 threshold; min, minutes.
[a] Norepinephrine tartrate

Table S2b Results of univariable analyses for 90-day mortality, additional time-intervals and parameters

	missing n	90-day survivors n=187	90-day non-survivors n=69	p
		**90-day mortality**		
**Mean arterial pressure (mmHg)**				
**0-6 h**	3	71.2 (66.9-76.5)	69.0 (62.6-72.8)	0.002
**0-24 h**	3	72.7 (68.9-76.6)	67.6 (62.5-73.0)	0.001
**Heart rate (bpm)**				
**0-6 h**	3	93.0 (77.4-107.1)	97.7 (80.9-114.5)	0.084
**0-24 h**	3	91.0 (77.6-102.6)	96.1 (83.4-109.9)	0.057
**Norepinephrine dose (µg/kg/min)[a]**				
**0-6 h**	4	0.17 (0.11-0.35)	0.32 (0.13-0.56)	<0.001
**0-24 h**	4	0.14 (0.07-0.30)	0.31 (0.13-0.60)	<0.001
**Arterial lactate (mmol/l)**				
**0-6 h**	13	2.2 (1.4-3.9)	4.2 (2.7-6.7)	<0.001
**0-24 h**	12	1.8 (1.2-3.0)	4.1 (2.4-6.6)	<0.001
**Capillary refill time (s)**				
**0-6 h**	2	5.2 (3.2-8.4)	7.4 (3.6-11.7)	0.022
**0-24 h**	0	5.0 (3.0-7.8)	8.5 (4.4-14.7)	<0.001
**Mottling score**				
**0-6 h**	1	0.0 (0.0-0.7)	0.4 (0.0-1.5)	<0.001
**0-24 h**	0	0.0 (0.0-0.3)	0.4 (0.0-1.6)	<0.001
**Peripheral temperature (°C)**				
**0-6 h**	6	31.9 (30.4-33.4)	31.5 (30.0-33.3)	0.118
**0-24 h**	1	32.6 (31.1-33.9)	32.2 (31.0-33.7)	0.284
**Central to peripheral temperature gradient (°C)**				
**0-6 h**	20	4.7 (3.1-6.6)	4.3 (2.6-6.1)	0.420
**0-24 h**	7	4.3 (2.8-5.8)	3.9 (2.5-5.7)	0.421
**Mean Peripheral StO2 (%)**				
**0-6 h**	4	65.2 (55.5-73.5)	61.3 (50.8-69.2)	0.027
**0-24 h**	2	68.4 (57.7-73.7)	61.8 (49.2-67.8)	<0.001
**AUT below StO_2 thresholds (%min)**				
**60 %**				
**0-6 h**	0	60.8 (0.0-1658.3)	343.0 (0.0-2939.9)	0.022
**0-24 h**	0	324.5 (0.0-6539.0)	2468.3 (236.3-11900.9)	<0.001
**50 %**				
**0-6 h**	0	0.0 (0.0-97.5)	0.0 (0.0-576.9)	0.053
**0-24 h**	0	0.0 (0.0-714.0)	304.8 (0.0-3775.1)	0.012
**40 %**				
**0-6 h**	0	0.0 (0.0-0.0)	0.0 (0.0-47.1)	0.026
**0-24 h**	0	0.0 (0.0-6.0)	0.0 (0.0-534.1)	0.008
**AOT above StO_2 80 % (%min)**				
**0-6 h**	0	0.0 (0.0-0.0)	0.0 (0.0-0.0)	0.107
**0-24 h**	0	0.0 (0.0-524.0)	0.0 (0.0-0.0)	<0.001
**Time below StO_2 thresholds (min)**				
**60 %**				
**0-6 h**	4	18.0 (0.0-211.8)	116.0 (0.0-287.0)	0.033
**0-24 h**	2	81.0 (0.0-813.0)	472.5 (55.5-880.1)	0.005
**50 %**				
**0-6 h**	4	0.0 (0.0-34.0)	0.0 (0.0-138)	0.082
**0-24 h**	2	0.0 (0.0-146.0)	36.8 (0.0-443.9)	0.015
**40 %**				
**0-6 h**	4	0.0 (0.0-0.0)	0.0 (0.0-6.5)	0.022
**0-24 h**	2	0.0 (0.0-1.1)	0.0 (0.0-115.0)	0.013
**Time above StO_2 80 % (min)**				

**0-6 h**	4	0.0 (0.0-0.3)	0.0 (0.0-0.0)	0.112
**0-24 h**	2	0.0 (0.0-102.8)	0.0 (0.0-0.0)	<0.001
**AUT below StO_2 thresholds divided by the length of follow-up (%)**				
**60 %**				
**0-6 h**				
**0-24 h**	0	0.24 (0.00-5.48)	1.07 (0.00-10.99)	0.016
**0-48 h**	0	0.25 (0.00-4.56)	1.89 (0.21-12.01)	<0.001
**50 %**	1	0.34 (0.00-4.01)	2.73 (0.42-11.28)	<0.001
**0-6 h**				
**0-24 h**	0	0.00 (0.00-0.29)	0.00 (0.00-1.70)	0.052
**0-48 h**	0	0.00 (0.00-0.53)	0.22 (0.00-3.64)	0.009
**40 %**	1	0.01 (0.00-0.66)	0.31 (0.00-4.37)	<0.001
**0-6 h**				
**0-24 h**	0	0.00 (0.00-0.00)	0.00 (0.00-0.15)	0.028
**0-48 h**	0	0.00 (0.00-0.05)	0.00 (0.00-0.45)	0.007
	1	0.00 (0.00-0.07)	0.01 (0.00-0.91)	<0.001
**AOT above StO_2 80 % divided by the length of follow-up (%)**				
**0-6 h**	0	0.00 (0.00-0.00)	0.00 (0.00-0.00)	0.106
**0-24 h**	0	0.00 (0.00-0.48)	0.00 (0.00-0.00)	<0.001
**0-48 h**	1	0.02 (0.00-0.71)	0.00 (0.00-0.01)	<0.001
**Time below StO_2 thresholds divided by the length of follow-up (%)**				
**60 %**				
**0-6 h**	4	6.4 (0.0-77.1)	48.1 (0.0-100.0)	0.020
**0-24 h**	2	7.8 (0.0-62.7)	35.3 (5.1-91.8)	0.001
**0-48 h**	1	6.8 (0.2-53.6)	37.7 (6.9-88.0)	<0.001
**50 %**				
**0-6 h**	4	0.0 (0.0-9.9)	0.0 (0.0-41.1)	0.082
**0-24 h**	2	0.0 (0.0-11.8)	2.7 (0.0-39.9)	0.010
**0-48 h**	1	0.1 (0.0-9.7)	8.1 (0.0-41.3)	<0.001
**40 %**				
**0-6 h**	4	0.0 (0.0-0.0)	0.0 (0.0-1.9)	0.023
**0-24 h**	2	0.0 (0.0-0.1)	0.0 (0.0-10.6)	0.011
**0-48 h**	1	0.0 (0.0-0.5)	0.1 (0.0-10.4)	<0.001
**Time above StO_2 80 % divided by the length of follow-up (%)**				
**0-6 h**	4	0.0 (0.0-0.1)	0.0 (0.0-0.0)	0.108
**0-24 h**	2	0.0 (0.0-8.7)	0.0 (0.0-0.0)	<0.001
**0-48 h**	1	0.9 (0.0-18.8)	0.0 (0.0-0.3)	<0.001

All continuous parameters are summarized as medians and interquartile ranges (IQR).
abbreviations: StO_2, tissue oxygen saturation; mmHg, millimeters of mercury; bpm, beats per minute; µg/kg/min, micrograms per kilogram per minute; mmol/l, millimoles per liter; s, seconds; °C, degrees of Celsius; AUT, area under StO_2 threshold; AOT, area over StO_2 threshold; min, minutes.
[a] Norepinephrine tartrate

Table S3a Results of AUROC analyses for organ dysfunction

Parameters	missing n	AUROC (95% CI)
**SOFA -score 0-24 h**	2	0.56 (0.48-0.64)
**SAPS II 0-24 h**	3	0.60 (0.52-0.68)
**APACHE 0-24 h**	3	0.56 (0.49-0.64)
**Mean arterial pressure (mmHg), 0-48 h ***	3	0.71 (0.64-0.78)
**Heart rate (bpm), 0-48 h**	3	0.57 (0.48-0.64)
**Norepinephrine dose (µg/kg/min), 0-48 h**	4	0.65 (0.57-0.73)
**Arterial lactate (mmol/l), 0-48 h**	12	0.68 (0.60-0.76)
**Capillary refill time (s), 0-48 h**	0	0.61 (0.53-0.68)
**Mottling score, 0-48 h**	0	0.58 (0.50-0.66)
**Peripheral temperature (°C), 0-48 h ***	1	0.57 (0.50-0.65)
**Central to peripheral temperature gradient (°C), 0-48 h**	6	0.50 (0.42-0.58)
**Mean Peripheral StO_2 (%), 0-48 h ***	1	0.59 (0.52-0.66)
**AUT below StO_2 thresholds (%min), 0-48 h**		
**60 %**	0	0.52 (0.45-0.60)
**50 %**	0	0.52 (0.44-0.59)
**40 %**	0	0.54 (0.46-0.62)
**AOT above StO_2 80 % (%min), 0-48 h ***	0	0.63 (0.56-0.70)
**Time below StO_2 thresholds (min), 0-48 h**		
**60 %**	1	0.51 (0.44-0.59)
**50 %**	1	0.51 (0.43-0.59)
**40 %**	1	0.53 (0.45-0.61)
**Time above StO_2 80 % (min), 0-48 h ***	1	0.62 (0.55-0.69)
**Logistic regression model 1**	12	0.78 (0.71-0.84)
**Logistic regression model 2**	12	0.78 (0.71-0.84)

Organ dysfunction was defined as unchanged or worsening SOFA score or death within 7 days in intensive care unit. Abbreviations: AUROC, Area Under Receiver Operating Characteristics Curve; SOFA, Sequential Organ Failure Assessment; h, hour; SAPS II, Simplified Acute Physiology Score; APACHE, Acute Physiology and Chronic Health Evaluation II; mmHg, millimeters of mercury; bpm, beats per minute; µg/kg/min, micrograms per kilogram per minute; mmol/l, millimoles per liter; s, seconds; °C, degrees of Celsius; StO_2, tissue oxygen saturation; AUT, area under StO_2 threshold; AOT, area over StO_2 threshold; min, minutes.
Variables in the logistic regression models; 48-hour mean capillary refill time; chronic kidney disease (moderate or severe) defined as glomerular filtration rate <60 ml/minute/1.73 m^2 or chronic dialysis at least one week prior to ICU admission; 48-hour mean lactate; 48-hour mean mean arterial pressure; 48-hour mean norepinephrine dose; 24-hour SAPS II score; smoking status (ever smoker); and in MODEL 1: peripheral 48-hour mean StO2; or in MODEL 2: 48-hour mean mottling score.
* In AUROC analysis smaller test result indicates more positive test.

Table S3b Results of AUROC analyses for 90-day mortality

Parameters	missing n	AUROC (95% CI)
**SOFA -score 0-24 h**	2	0.76 (0.69-0.82)
**SAPS II 0-24 h**	3	0.80 (0.78-0.85)
**APACHE 0-24 h**	3	0.71 (0.64-0.78)
**Mean arterial pressure (mmHg), 0-48 h ***	3	0.75 (0.68-0.82)
**Heart rate (bpm), 0-48 h**	3	0.62 (0.54-0.79)
**Norepinephrine dose (µg/kg/min), 0-48 h**	4	0.72 (0.65-0.80)
**Arterial lactate (mmol/l), 0-48 h**	12	0.82 (0.76-0.88)
**Capillary refill time (s), 0-48 h**	0	0.71 (0.63-0.79)
**Mottling score, 0-48 h**	0	0.72 (0.65-0.80)
**Peripheral temperature (°C), 0-48 h ***	1	0.59 (0.51-0.67)
**Central to peripheral temperature gradient (°C), 0-48 h**	6	0.50 (0.41-0.58)
**Mean Peripheral StO_2 (%), 0-48 h ***	1	0.67 (0.60-0-75)
**AUT below StO_2 thresholds (%min), 0-48 h**		
**60 %**	0	0.65 (0.57-0.72)
**50 %**	0	0.63 (0.55-0.71)
**40 %**	0	0.62 (0.54-0.70)
**AOT above StO_2 80 % (%min), 0-48 h ***	0	0.68 (0.61-0.75)
**Time below StO_2 thresholds (min), 0-48 h**		
**60 %**	1	0.62 (0.55-0.70)
**50 %**	1	0.62 (0.54-0.70)
**40 %**	1	0.62 (0.54-0.70)
**Time above StO_2 80 % (min), 0-48 h ***	1	0.68 (0.60-0.75)
**Logistic regression model 1**	13	0.87 (0.83-0.92)
**Logistic regression model 2**	12	0.89 (0.84-0.93)

AUROC, Area Under Receiver Operating Characteristics Curve; SOFA, Sequential Organ Failure Assessment; h, hour; SAPS II, Simplified Acute Physiology Score; APACHE, Acute Physiology and Chronic Health Evaluation II; mmHg, millimeters of mercury; bpm, beats per minute; µg/kg/min, micrograms per kilogram per minute; mmol/l, millimoles per liter; s, seconds; °C, degrees of Celsius; StO_2, tissue oxygen saturation; AUT, area under StO_2 threshold; AOT, area over StO_2 threshold; min, minutes.
Variables in the logistic regression models; 48-hour mean capillary refill time; chronic kidney disease (moderate or severe) defined as glomerular filtration rate <60 ml/minute/1.73 m^2 or chronic dialysis at least one week prior to ICU admission; 48-hour mean lactate; 48-hour mean mean arterial pressure; 48-hour mean norepinephrine dose; 24-hour SAPS II score; smoking status (ever smoker); and in MODEL 1: peripheral 48-hour mean StO2; or in MODEL 2: 48-hour mean mottling score.
* In AUROC analysis smaller test result indicates more positive test.

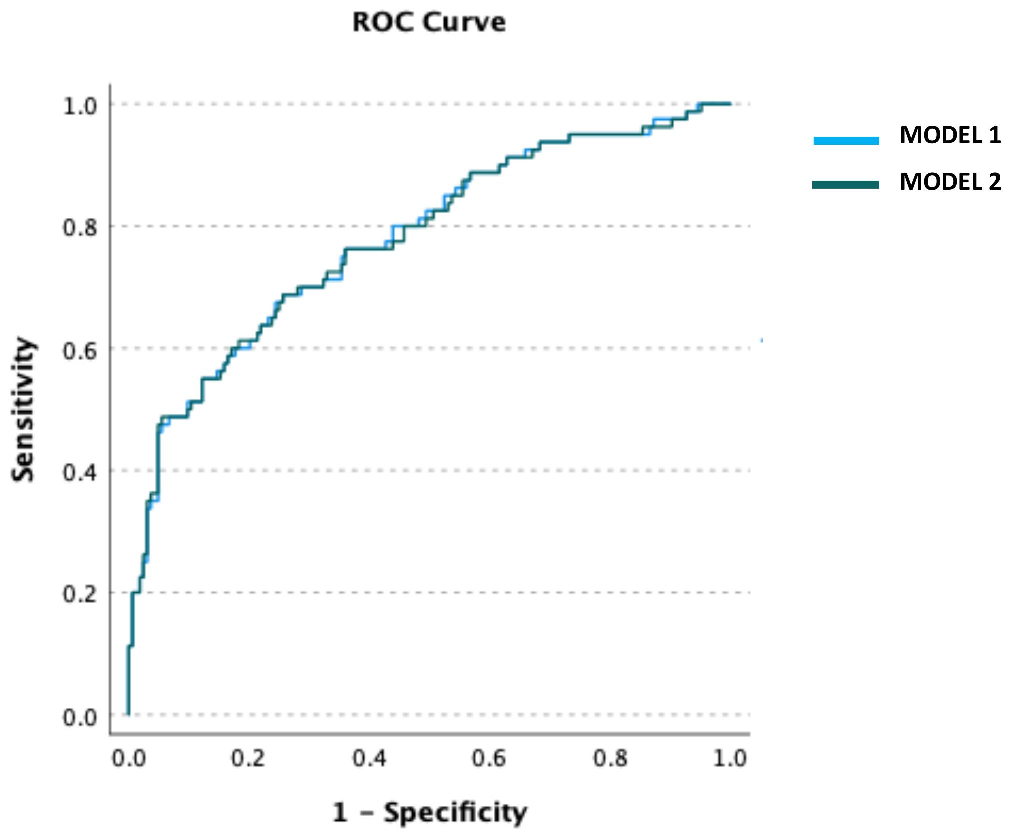

Fig. S5a ROC curves of logistic regression models at predicting organ dysfunction. Organ dysfunction was defined as unchanged or worsening SOFA score or death within 7 days in intensive care unit. Variables in the model; 48-hour mean capillary refill time; chronic kidney disease (moderate or severe) defined as glomerular filtration rate <60 ml/minute/1.73 m^2 or chronic dialysis at least one week prior to ICU admission; 48-hour mean lactate; 48-hour mean mean arterial pressure; 48-hour mean norepinephrine dose; 24-hour SAPS II score; smoking status (ever smoker); and in MODEL 1: peripheral 48-hour mean StO2; or in MODEL 2: 48-hour mean mottling score.
Abbreviations SAPS II, Simplified Acute Physiology Score, StO2, Tissue Oxygen Saturation

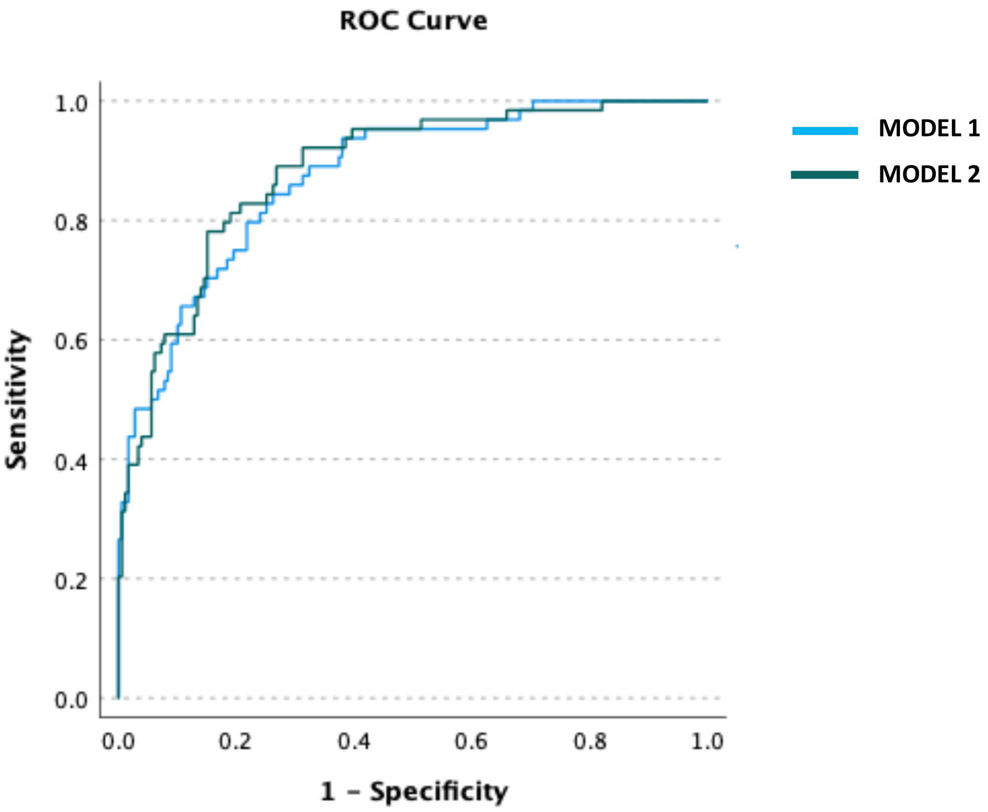

Fig. S5b ROC curves of logistic regression models at predicting 90-day mortality. Variables in the model; 48-hour mean capillary refill time; chronic kidney disease (moderate or severe) defined as glomerular filtration rate <60 ml/minute/1.73 m^2 or chronic dialysis at least one week prior to ICU admission; 48-hour mean lactate; 48-hour mean mean arterial pressure; 48-hour mean norepinephrine dose; 24-hour SAPS II score; smoking status (ever smoker); and in MODEL 1: peripheral 48-hour mean StO2; or in MODEL 2: 48-hour mean mottling score.
Abbreviations SAPS II, Simplified Acute Physiology Score, StO2, Tissue Oxygen Saturation

Table S4 Early versus late deaths

	Missing n	Early deaths (Within 48 h) n=29	Missing n	Late deaths (48 h to 90 d) n=40	p
Age (years)	0	67.0 (61.5; 74.0)	0	70.0 (61.0; 75.0)	0.543
Gender (female)	0	15 (51.7)	0	25 (62.5)	0.461
Total Charlson Comorbidity Index score	0	4.0 (3.0; 6.0)	0	6.0 (3.0; 7.0)	0.062
Smoking (ever)	0	17 (58.6)	0	25 (62.5)	0.806
Invasive mechanical ventilation	0	27 (93.1)	0	36 (90.0)	1.000
Norepinephrine dose at enrolment	0	0.46 (0.28; 0.75)	0	0.23 (0.12; 0.33)	0.001
Lactate at enrolment	4	5.6 (3.7; 13.7)	4	3.5 (2.1; 5.4)	<0.001
Sepsis diagnosis at enrolment	0	24 (82.8)	0	34 (85.0)	1.000
Septic shock diagnosis at enrolment	0	24 (82.8)	0	27 (67.5)	0.177
AKI during days 1-7	0	28 (96.6)	0	33 (82.5)	0.126
RRT (CRRT or IHD or both) during days 1-7	0	20 (69.0)	0	15 (37.5)	0.015
SOFA 24h	2	13.0 (12.0; 16.0)	0	11.5 (9.0; 14.0)	0.007
SOFA max	2	15.0 (13.0; 16.0)	0	13.5 (9.3; 15.0)	0.092
SAPS II	1	71.5 (58.0; 79.3)	0	53.5 (46.0; 66.0)	<0.001
APACHE	1	32.5 (27.0; 37.8)	0	26.0 (22.3; 34.0)	0.027
length of ICU stay (days)	0	1.4 (1.0; 1.6)	0	6.7 (2.9; 10.4)	<0.001
Treatment limitations	0	5 (17.2)	40		
Decision to withdraw life sustaining therapy due to treatment failure	0	25 (86.2)	40		
Mean arterial pressure (mmHg)	1		0		
48-h mean		62.7 (58.4; 67.1)		71.0 (68.7; 76.5))	<0.001
48-h mean, 6 hours pre-death excluded		64.9 (59.9;67.2)			
Norepinephrine dose (µg/kg/min)	1		0		
48-h mean		0.41 (0.18; 0.65)		0.19 (0.07; 0.39)	0.023
48-h mean, 6 hours pre-death excluded		0.60 (0.42; 0.98)			
Arterial lactate (mmol/l)	2		2		
48-h mean		7.5 (5.1; 11.2)		2.6 (1.8; 4.3)	<0.001
48-h mean, 6 hours pre-death excluded		7.5 (4.6; 11.2)			
Capillary refill time (s)	0		0		
48-h mean		13.1 (5.7; 21.9)		7.1 (3.3; 13.5)	0.031
48-h mean, 6 hours pre-death excluded		11.8 (5.2; 20.1)			
Mottling score	0		0		
48-h mean		0.6 (0.0; 1.9)		0.26 (0.0; 1.5)	0.396
48-h mean, 6 hours pre-death excluded		0.5 (0.0; 1.6)			
Mean Peripheral StO_2 (%)	1		0		
48-h mean		57.1 (47.9; 64.7)		64.8 (52.3; 69.4))	0.090
48-h mean, 6 hours pre-death excluded		59.1 (48.2; 66.3)			

All continuous parameters are summarized as medians and interquartile ranges (IQR), categorical variables as counts and percentages.

Early deaths occurred within the 48-hour StO_2 monitoring period. Late deaths occurred later than 48 hours but within 90-days from study enrollment.

Abbreviations: SOFA, Sequential Organ Failure Assessment; Chronic Kidney Disease defined as glomerular filtration rate (GFR) <60 ml/minute/1.73 m2 or chronic dialysis at least one week prior to ICU admission; APACHE, Acute Physiology and Chronic Health Evaluation; Sepsis and septic shock defined according to Sepsis-3 [17]; AKI, Acute Kidney Injury, defined by Kidney Disease Improving Global Outcomes (KDIGO) Clinical Practice Guidelines [18]; RRT, Renal Replacement Therapy; CRRT, Continuous Renal Replacement Therapy; IHD, Intermittent Hemodialysis; SAPS II, Simplified Acute Physiology Score; ICU, Intensive Care Unit; StO2, tissue oxygen saturation; mmHg, millimeters of mercury; bpm, beats per minute; µg/kg/min, micrograms per kilogram per minute; mmol/l, millimoles per liter; s, seconds; °C, degrees of Celsius; AUT, area under StO_2 threshold; AOT, area over StO_2 threshold; min, minutes.

Table S5 Results of univariable analyses for organ dysfunction and 90-day mortality excluding data from the last six hours preceding the death in patients, who died within the 48-hour StO_2 monitoring period

		**Organ dysfunction**			**90-day mortality**		
	**missing n**	**SOFA-improvers n=171**	**SOFA-non-improvers n=85**	**p**	**Survivors n=187**	**Non-survivors n=69**	**p**
Mean arterial pressure (mmHg)[a]	3	74.5 (70.2; 78.2)	69.4 (65.3; 74.4)	<0.001	73.9 (70.2; 78.0)	68.9 (63.1; 73.5)	<0.001
Norepinephrine dose (µg/kg/min)[a]	4	0.10 (0.05; 0.24)	0.25 (0.07; 0.54)	<0.001	0.10 (0.05; 0.23)	0.32 (0.13; 0.61)	<0.001
Arterial lactate (mmol/l)[a]	12	1.6 (1.1; 2.4)	2.9 (1.4; 5.4)	<0.001	1.5 (1.1; 2.3)	4.1 (2.2; 6.2)	<0.001
Capillary refill time (s)[a]	0	4.5 (2.8; 7.4)	6.3 (3.2; 12.3)	0.010	4.3 (2.8; 7.2)	9.3 (3.9; 14.6)	<0.001
Mottling score[a]	0	0.0 (0.0; 0.3)	0.1 (0.0; 0.9)	0.029	0.0 (0.0; 0.2)	0.4 (0.0; 1.5)	<0.001
Mean Peripheral StO_2 (%)[a]	1	68.3 (57.5; 74.1)	64.7 (53.4; 70.8)	0.044	68.7 (58.2; 74.5)	61.8 (51.2; 67.4)	<0.001

All continuous parameters are summarized as medians and interquartile ranges (IQR).
Organ dysfunction was defined as unchanged or worsening SOFA score or death within 7 days in intensive care unit.
Abbreviations: StO_2, tissue oxygen saturation; SOFA, Sequential Organ Failure Assessment; h, hour; SOFA; improvers, patients whose SOFA score decreased during the 7 day follow-up; SOFA-non-improvers, patients whose SOFA score stayed the same or increased, or who died during the 7-day follow-up; mmHg, millimeters of mercury; bpm, beats per minute; µg/kg/min, micrograms per kilogram per minute; mmol/l, millimoles per liter; s, seconds; °C, degrees of Celsius; min, minutes.
[a] 48-h mean, 6 hours pre-death excluded, for the patients who died within the 48-hour StO_2 monitoring period

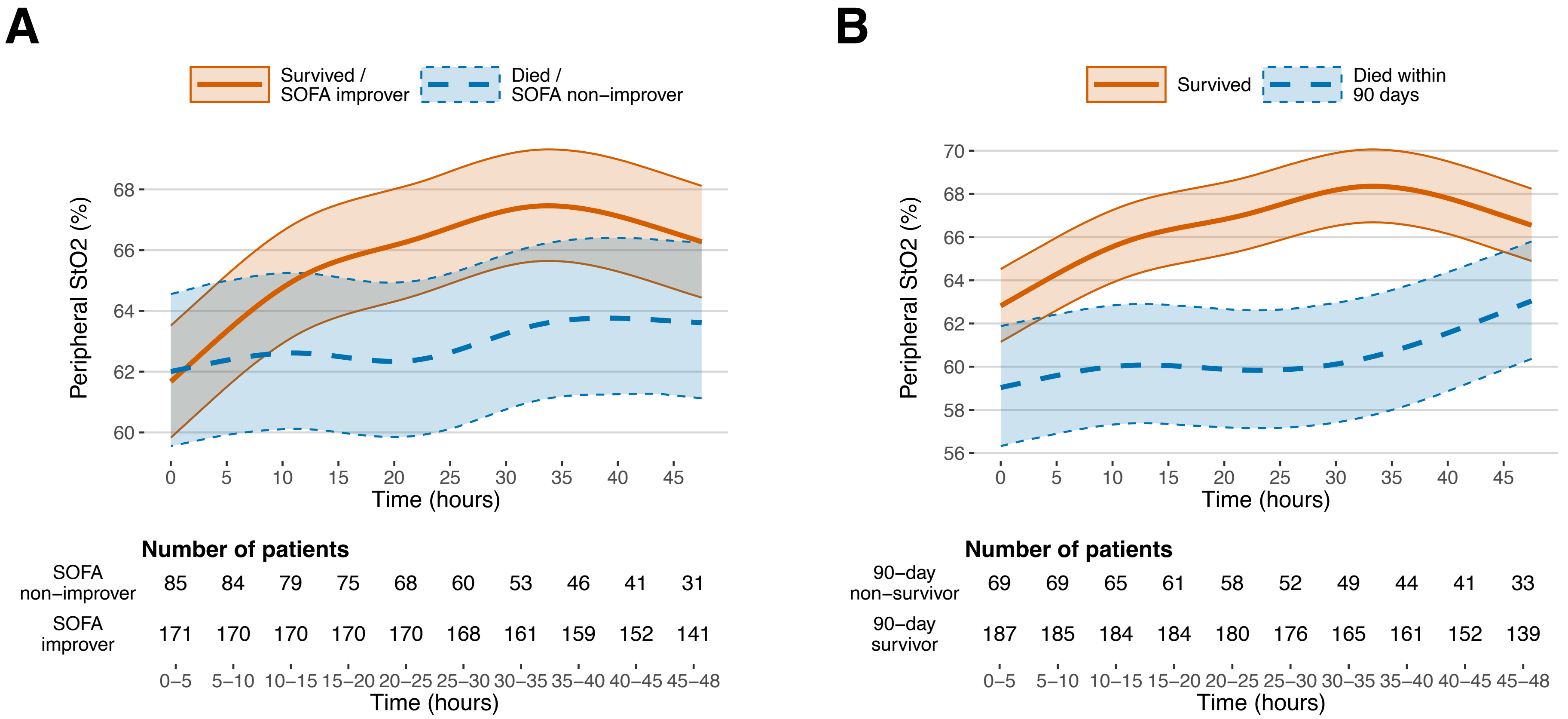

Fig. S6a and S6b Mean StO_2 and organ dysfunction and mean StO_2 and 90-day mortality estimated using linear mixed models. The last six hours of the StO_2 registration was excluded in the patients who died during the registration. Time was modeled using restricted cubic splines with 5 knots and interaction with organ dysfunction (Figure 6a) or 90-day mortality (Figure 6b). The shaded areas represent the 95% confidence interval. SOFA-improvers were patients, whose SOFA score decreased during the 7 day follow-up, and SOFA-non-improvers patients, whose SOFA score stayed the same or increased, or who died during the 7-day follow-up.

Table S6a Sensitivity, specificity, positive and negative predictive values, and likelihood ratios in predicting organ dysfunction

**Parameter**	**Cut-off**	**Se** (%)	**95% CI** (%)	**Sp** (%)	**95% CI** (%)	**NPV** (%)	**95% CI** (%)	**PPV** (%)	**95% CI** (%)	**+LR**	**95% CI**	**-LR**	**95% CI**
**StO_2 (%)** 0-48 h	60*	41.7	31.0-52.9	70.8	63.3-77.5	71.2	66.8-75.2	41.2	33.2-49.7	1.43	1.01-2.01	0.82	0.67-1.01
	50*	17.9	10.4-27.7	87.1	81.2-91.8	68.4	65.8-70.8	40.5	27.2-55.5	1.39	0.76-2.53	0.94	0.84-1.06
	40*	4.8	1.3-11.8	98.3	95.0-99.6	67.7	66.6-68.9	57.1	23.4-85.3	2.71	0.62-11.85	0.97	0.92-1.02
	80*	94.1	86.7-98.0	8.2	4.6-13.4	73.7	51.1-88.3	33.5	31.9-35.1	1.02	0.96-1.10	0.73	0.27-1.95
	66.5*	59.5	48.3-70.1	55.0	47.2-62.6	73.4	67.4-78.7	39.4	33.8-45.3	1.32	1.04-1.68	0.74	0.55-0.99
**MAP (mmHg)** 0-48 h	72.5*	69.1	58.0-78.7	60.4	52.6-67.8	79.7	73.6-84.7	46.4	40.6-52.3	1.74	1.38-2.20	0.51	0.36-0.72
**HR (bpm)** 0-48 h	91.7	57.1	45.9-67.9	54.4	46.6-62.1	71.9	65.8-77.2	38.4	32.7-44.4	1.25	0.98-1.61	0.79	0.59-1.04
**Norepinephrine dose (µg/kg/min),** 0-48 h	0.13	66.3	55.1-76.3	57.4	49.6-65.0	77.6	71.4-82.8	43.3	37.7-49.1	1.56	1.23-1.96	0.59	0.42-0.82
**Lactate (mmol/l)**, 0-48 h	1.8	63.8	52.2-74.2	56.7	48.8-64.4	76.2	70.0-81.5	41.8	36.1-47.8	1.47	1.16-1.87	0.64	0.46-0.88
**CRT (s)**, 0-48 h	5.0	62.4	51.2-72.6	56.7	48.9-64.3	75.2	69.1-80.4	41.7	36.1-47.6	1.44	1.14-1.83	0.66	0.49-0.90
**Mottling score**, 0-48 h	0.0	54.1	43.0-65.0	52.6	44.9-60.3	69.8	63.8-75.2	36.2	30.6-42.2	1.14	0.89-1.47	0.87	0.66-1.14

Organ dysfunction was defined as unchanged or worsening SOFA score or death within 7 days in intensive care unit.
Apart from the StO_2 cut-offs (60%, 50%, 40% and 80%), we did not predefine the cut-offs for other parameters. Therefore we chose to use median values calculated for the whole cohort (n=256) as cut-offs for different clinical parameters. An online calculator (https://www.medcalc.org/calc/diagnostic_test.php) served to determine these properties of diagnostic accuracy.
Abbreviations: 95% CI, 95% confidence Interval; Se, sensitivity; Sp, Specificity; NPV, negative predictive value; PPV, positive predictive value; +LR, positive likelihood ratio; -LR, negative likelihood ratio; StO_2, tissue oxygen saturation; MAP, mean arterial pressure; HR, heart rate; CRT, capillary refill time; mmHg, millimeters of mercury; bpm, beats per minute; µg/kg/min, micrograms per kilogram per minute; mmol/l, millimoles per liter; s, seconds.
* values under the cut-off indicate positive test

Table S6b Sensitivity, specificity, positive and negative predictive values, and likelihood ratios in predicting death at 90-days

Parameter	Cut-off	Se (%)	95% CI (%)	Sp (%)	95% CI (%)	NPV (%)	95% CI (%)	PPV (%)	95% CI (%)	+LR	95% CI	-LR	95% CI
**StO_2 (%)** 0-48 h	60*	47.1	34.8-59.6	71.7	64.6-78.0	78.8	74.5-82.6	37.7	30.1-45.9	1.66	1.18-2.33	0.74	0.58-0.94
	50*	26.5	16.5-38.6	89.8	84.6-93.8	77.1	74.3-79.6	48.7	34.6-62.9	2.61	1.46-4.66	0.82	0.70-0.95
	40*	5.9	1.6-14.4	98.4	95.4-99.7	74.2	73.0-75.4	57.1	23.5-85.3	3.67	0.84-15.96	0.96	0.90-1.02
	80*	95.6	87.6-99.1	7.6	4.2-12.4	82.4	58.1-94.0	27.5	26.3-28.9	1.03	0.97-1.10	0.58	0.17-1.97
	66.5*	70.6	58.3-81.0	57.8	50.3-64.9	84.4	78.6-88.8	37.8	32.6-43.3	1.67	1.33-2.10	0.51	0.35-0.75
**MAP (mmHg)** 0-48 h	72.5*	73.5	61.4-83.5	59.5	52.0-66.6	85.9	80.2-90.2	40.0	34.7-45.5	1.81	1.45-2.27	0.45	0.29-0.67
**HR (bpm)** 0-48 h	91.7	66.2	53.7-77.2	56.8	49.3-64.0	82.0	76.2-86.7	36.0	30.7-41.6	1.53	1.21-1.94	0.60	0.42-0.85
**Norepinephrine dose (µg/kg/min),** 0-48 h	0.13	75.0	63.0-84.7	58.7	51.2-65.9	86.4	80.5-90.7	40.2	35.0-45.5	1.82	1.46-2.26	0.43	0.28-0.65
**Lactate (mmol/l)**, 0-48 h	1.8	84.6	73.5-92.4	62.6	55.0-69.7	91.8	86.2-95.2	45.1	39.8-50.5	2.26	1.82-2.81	0.25	0.14-0.44
**CRT (s)**, 0-48 h	5.0	71.0	58.8-81.3	58.3	50.9-65.4	84.5	78.7-88.9	38.6	33.4-44.1	1.70	1.36-2.14	0.50	0.34-0.73
**Mottling score**, 0-48 h	0.0	71.0	58.8 -81.3	58.3	50.9 -65.4	84.5	78.7 -88.9	38.6	33.4 -44.1	1.70	1.36-2.14	0.50	0.34-0.73

Apart from the StO_2 cut-offs (60%, 50%, 40% and 80%), we did not predefine the cut-offs for other parameters. Therefore we chose to use median values calculated for the whole cohort (n=256) as cut-offs for different clinical parameters. An online calculator (https://www.medcalc.org/calc/diagnostic_test.php) served to determine these properties of diagnostic accuracy. Abbreviations: 95% CI, 95% confidence Interval; Se, sensitivity; Sp, Specificity; NPV, negative predictive value; PPV, positive predictive value; +LR, positive likelihood ratio; -LR, negative likelihood ratio; StO_2, tissue oxygen saturation; MAP, mean arterial pressure; HR, heart rate; CRT, capillary refill time; mmHg, millimeters of mercury; bpm, beats per minute; µg/kg/min, micrograms per kilogram per minute; mmol/l, millimoles per liter; s, seconds.
* values under the cut-off indicate positive test

Table S7 Results of logistic regression models for organ dysfunction and 90-day mortality excluding data from the last six hours preceding the death in patients, who died within the 48-hour StO_2 monitoring period

	Organ dysfunction		90-day mortality	
**Variables in the model**	**Odds ratio (95% Confidence interval)**	**p**	**Odds ratio (95% Confidence interval)**	**p**
**MODEL 1**				
Capillary refill time[a]	-	ns	-	ns
Chronic kidney disease	-	ns	2.82 (1.06-7.52)	0.038
Lactate[a]	1.28 (1.10-1.48)	0.001	1.30 (1.10-1.54)	0.002
Mean arterial pressure[a]	0.92 (0.87-0.97)	0.002	0.92 (0.86-0.98)	0.007
Norepinephrine dose[a]	-	ns	-	ns
Peripheral StO_2[a]	-	ns	-	ns
SAPS II score[b]	-	ns	1.05 (1.02-1.08)	<0.001
Smoker (ever)	2.52 (1.35-4.69)	0.004	2.70 (1.28-5.69)	0.009
**MODEL 2**				
Capillary refill time[a]	-	ns	-	ns
Chronic kidney disease	-	ns	3.11 (1.16-8.32)	0.024
Lactate[a]	1.27 (1.10-1.47)	0.001	1.28 (1.09-1.52)	0.004
Mean arterial pressure[a]	0.92 (0.87-0.97)	0.002	0.92 (0.86-0.99)	0.017
Mottling score[a]	-	ns	1.82 (1.09-3.04)	0.022
Norepinephrine dose[a]	-	ns	-	ns
SAPS II score[b]	-	ns	1.04 (1.02-1.07)	0.002
Smoker (ever)	2.37 (1.28-4.37)	0.006	2.43 (1.13-5.22)	0.023

Binary logistic regression models (forward stepwise, conditional). Model 1 contains peripheral 48-hour mean StO_2 and Model 2 contains 48-hour mean mottling score instead.
Organ dysfunction was defined as unchanged or worsening SOFA score or death within 7 days in intensive care unit. Chronic kidney disease (moderate or severe) was defined as glomerular filtration rate <60 ml/minute/1.73 m^2 or chronic dialysis at least one week prior to ICU admission.
Abbreviations: SAPS II, Simplified Acute Physiology Score; MAP, Mean Arterial Pressure; StO_2, Tissue Oxygen Saturation; ns, not significant
[a] 48-hour mean, 6 hours pre-death excluded for the patients, who died within the 48-hour StO_2 monitoring period
[b] 0-24 hours

Table S8 Results of univariable and adjusted logistic regression models for delta variables within the first 48 hours and outcomes

		Organ dysfunction				90-day mortality			
		Univariable		Adjusted		Univariable		Adjusted	
**Delta variable**	**Mean (IQR)**	**Odds ratio (95% CI)**	**p**	**Odds ratio (95% CI)**	**p**	**Odds ratio (95% CI)**	**p**	**Odds ratio (95% CI)**	**p**
Delta capillary refill time[a]	-1.0 (-4.0; 1.0)	1.03 (1.01 - 1.06)	0.008	1.00 (0.97 - 1.02)	0.829	1.07 (1.03 - 1.10)	0.000	1.03 (1.00 - 1.07)	0.081
Delta lactate[a]	-1.1 (-2.5; -0.3)	1.25 (1.13 - 1.40)	0.000	1.03 (0.92 - 1.18)	0.628	1.25 (1.13 - 1.41)	0.000	1.03 (0.92 - 1.19)	0.621
Delta mean arterial pressure[b]	3.9 (-5.1; 14.2)	0.95 (0.93 - 0.96)	0.000	0.95 (0.93 - 0.98)	0.000	0.94 (0.92 - 0.96)	0.000	0.95 (0.93 - 0.98)	0.000
Delta norepinephrine dose[b]	-0.10 (-0.22; -0.03)	59.13 (14.69 - 298.97)	0.000	15.86 (2.99 - 99.53)	0.002	42.37 (11.22 - 196.13)	0.000	8.34 (1.61 - 49.91)	0.015
Delta mottling score[a]	0.0 (0.0; 0.0)	1.50 (1.12 - 2.07)	0.009	1.00 (0.68 - 1.50)	0.984	1.63 (1.19 - 2.33)	0.004	1.02 (0.69 - 1.55)	0.939
Delta peripheral StO_2[b]	1.8 (-5.6; 8.4)	0.95 (0.92 - 0.97)	0.000	0.95 (0.92 - 0.98)	0.005	0.95 (0.93 - 0.98)	0.000	0.98 (0.95 - 1.01)	0.297

Organ dysfunction was defined as unchanged or worsening SOFA score or death within 7 days in intensive care unit.
Abbreviations: IQR, inter quartile range; 95% CI, 95% confidence interval
[a] Delta value calculated as a difference of the first and the last observation within the first 48 hours
[b] Delta value calculated as a difference of the means of the first and the last observation hours within the first 48 hour

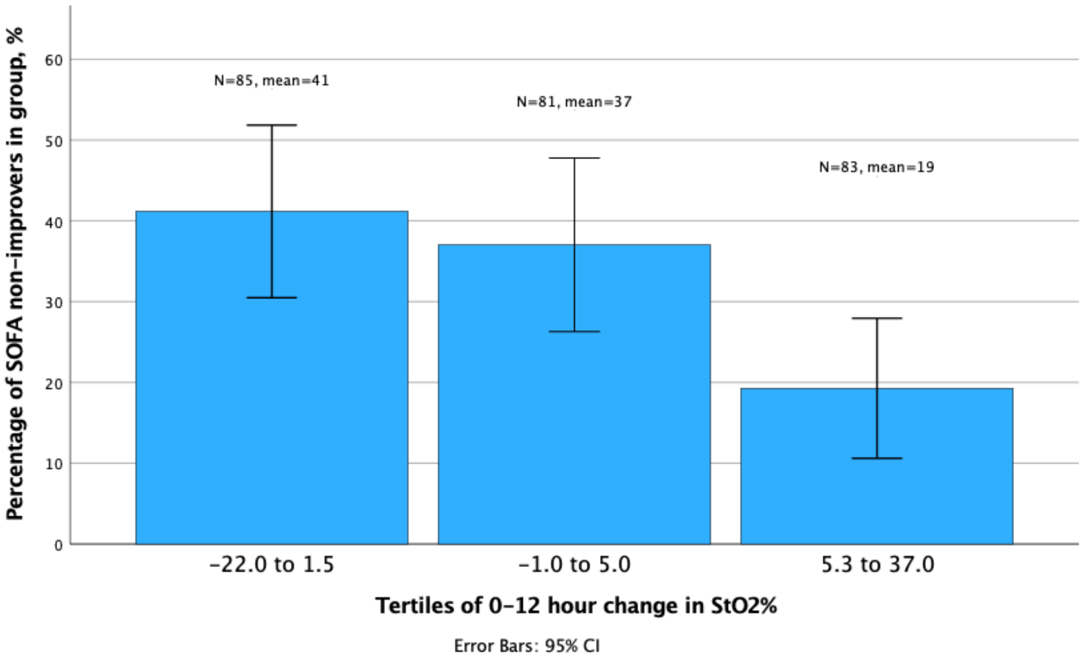

Fig. S7a Tertiles of delta StO_2 over the first 12 hours and organ dysfunction. SOFA-non-improvers were patients whose SOFA score stayed the same or increased, or who died during the 7-day follow-up in ICU. Delta StO_2 values were calculated as a difference of the median of the first and the 12th observation hour.

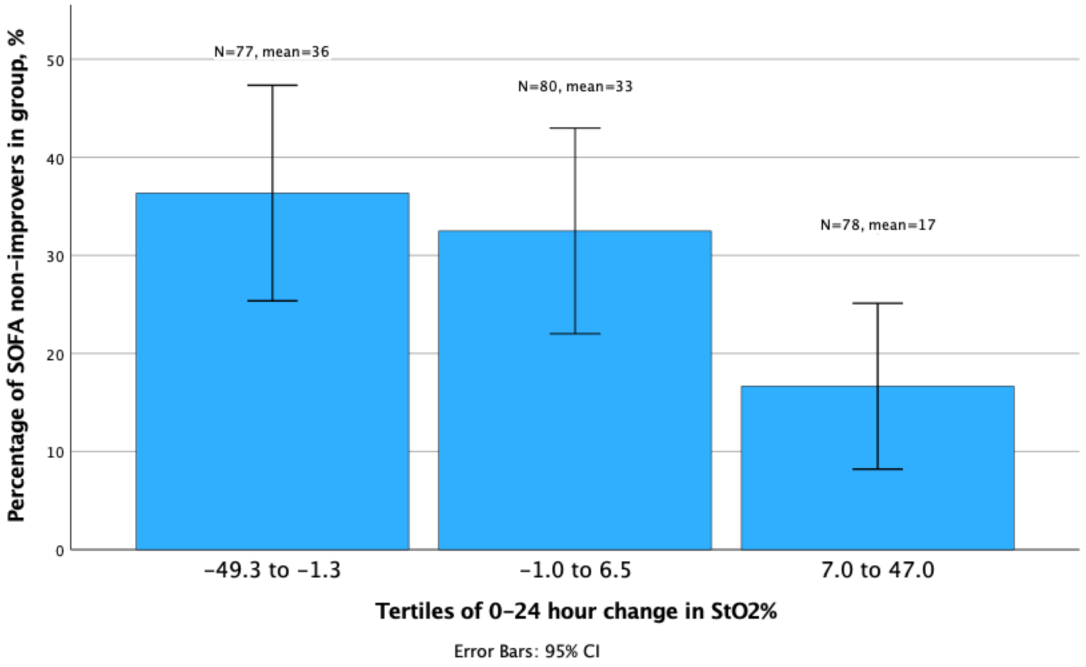

Fig. S7b Tertiles of delta StO_2 over the first 24 hours and organ dysfunction. SOFA-non-improvers were patients whose SOFA score stayed the same or increased, or who died during the 7-day follow-up in ICU. Delta StO_2 values were calculated as a difference of the median of the first and the 24th observation hour. The odds ratio for the 24-hour delta StO_2 for organ dysfunction was 0.95 (95% confidence interval 0.92-0.98, p=0.001) and 0.96 (95% confidence interval 0.92-0.98, p=0.004) when excluding the last 6 hours of data in the patients, who died within the first 48 hours.

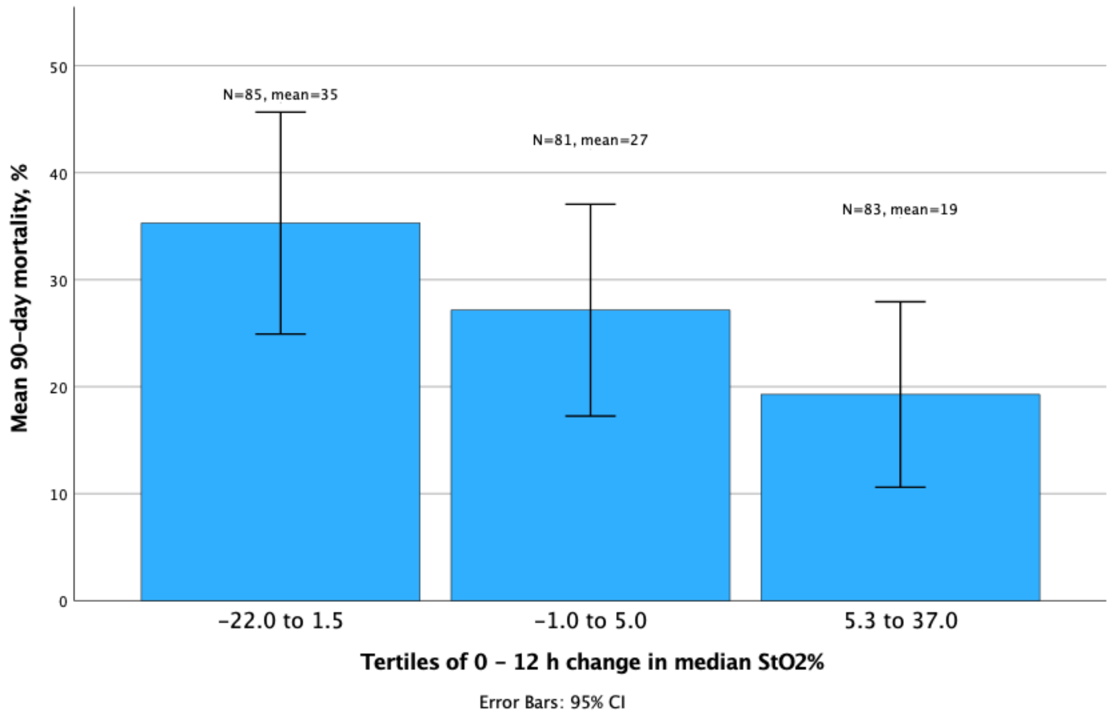

Fig. S7c Tertiles of delta StO_2 over the first 12 hours and 90-day mortality. SOFA-non-improvers were patients whose SOFA score stayed the same or increased, or who died during the 7-day follow-up in ICU. Delta StO_2 values were calculated as a difference of the median of the first and the 12th observation hour.

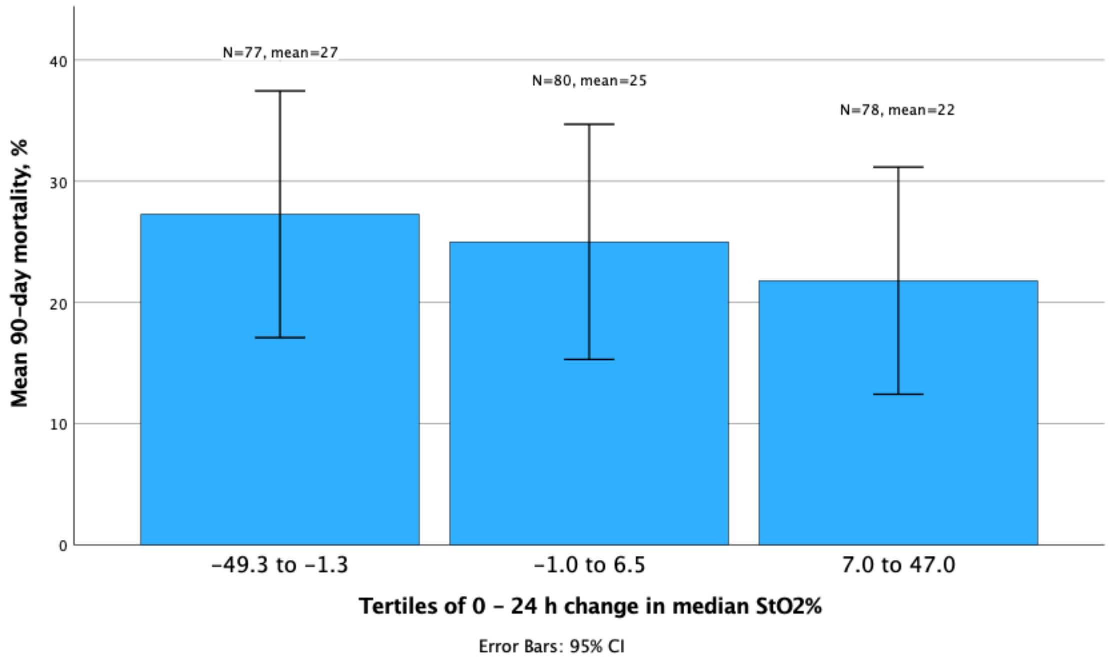

Fig. S7d Tertiles of delta StO_2 over the first 24 hours and 90-day mortality. SOFA-non-improvers were patients whose SOFA score stayed the same or increased, or who died during the 7-day follow-up in ICU. Delta StO_2 values were calculated as a difference of the median of the first and the 24th observation hour. The odds ratio for the 24-hour delta StO_2 for 90-day mortality was 0.97 (95% confidence interval 0.94-0.99, p=0.022) and 0.97 (95% confidence interval 0.94-1.00, p=0.076) when excluding the last 6 hours of data in the patients, who died within the first 48 hours.

Table S9 Patient characteristics of sepsis sub-group

			Organ dysfunction			90-day mortality		
	**Missing n**	**All patients n=206**	**SOFA-improvers n=139**	**SOFA-non-improvers n= 67**	**p**	**Survivors n=148**	**Non-survivors n=58**	**p**
Age (years)	0	66.0 (57.0; 72.0)	67.0 (56.0; 73.0)	64 (58.0; 70.0)	0.409	64.0 (54.0; 72.0)	67.5 (61.0; 73.0)	0.070
Gender (female)	0	92 (44.7)	57 (41.0)	35 (52.2)	0.137	66 (44.6)	26 (44.8)	1.000
Co-morbidity								
Total Charlson Comorbidity Index score	0	4.0 (2.0; 6.0)	4.0 (2.0; 6.0)	4.0 (2.0; 6.0)	0.882	4.0 (2.0; 5.0)	5.0 (3.0; 7.0)	0.002
Diabetes mellitus	0	65 (31.6)	41 (29.5)	24 (35.8)	0.424	42 (28.4)	23 (39.7)	0.135
Peripheral or cerebral arterial disease	0	43 (20.9)	29 (20.9)	14 (20.9)	1.000	27 (18.2)	16 (27.6)	0.181
Coronary artery disease	0	21 (10.2)	16 (11.5)	95(7.5)	0.465	13 (8.8)	8 (13.8)	0.310
Hypertension	0	121 (58.7)	84 (60.4)	37 (55.2)	0.546	83 (56.1)	38 (65.5)	0.271
Chronic kidney disease	0	27 (13.1)	17 (12.2)	10 (14.9)	0.661	16 (10.8)	11 (19.0)	0.166
Chronic heart failure	0	16 (7.8)	12 (8.6)	4 (6.0)	0.590	12 (8.1)	4 (6.9)	1.000
Smoking (ever)	0	95 (46.1)	53 (38.1)	42 (62.7)	0.001	60 (40.5)	35 (60.3)	0.013
APACHE IV diagnostic group	0							
cardiovascular (non-operative)		58 (28.2)	38 (27.3)	20 (29.9)	0.934	44 (29.7)	14 (24.1)	0.783
respiratory tract (non-operative)		17 (8.3)	13 (9.4)	4 (6.0)		11 (7.4)	6 (10.3)	
gastrointestinal tract (non-operative)		13 (6.3)	9 (6.5)	4 (6.0)		9 (6.1)	4 (6.9)	
gastrointestinal tract (operative)		79 (38.3)	56 (40.3)	23 (34.3)		55 (37.2)	24 (41.4)	
other (<5% each)		37 (18.0)	23 (16.5)	16 (23.8)		29 (19.6)	10 (17.3)	
Invasive mechanical ventilation	0	157 (76.2)	106 (76.3)	51 (76.1)	1.000	105 (70.9)	52 (89.7)	0.004
Norepinephrine dose at enrolment	0	0.22 (0.12; 0.39)	0.20 (0.11; 0.33)	0.28 (0.16; 0.49)	0.010	0.20 (0.11; 0.33)	0.33 (0.16; 0.56)	0.002
Lactate at enrolment	20	2.5 (1.5; 4.8)	2.2 (1.4; 3.7)	3.9 (1.6; 6.0)	0.004	2.2 (1.3; 3.6)	4.5 (2.9; 8.4)	<0.001
Dominant shock type	3							
Distributive		185 (91.1)	126 (90.6)	59 (92.2)	1.000	132 (90.4)	53 (93.0)	1.000
Cardiogenic		5 (2.5)	4 (2.9)	1 (1.6)		4 (2.7)	1 (1.8)	
Hypovolemic		13 (6.4)	9 (6.5)	4 (6.3)		10 (6.8)	3 (5.3)	
Obstructive		0	0	0		0	0	
Septic shock diagnosis at enrolment	0	151 (73.3)	98 (70.5)	53 (79.1)	0.240	100 (67.6)	51 (87.9)	0.003
AKI during days 1-7	0	130 (63.1)	79 (56.8)	51 (76.1)	0.009	79 (53.4)	51 (87.9)	<0.001
RRT (CRRT or IHD or both) during days 1-7	0	61 (29.6)	27 (19.4)	34 (50.7)	<0.001	32 (21.6)	29 (50.0)	<0.001
SOFA 24h	1	9.0 (7.0; 13.0)	9.0 (7.0; 12.0)	10.0 (7.0; 13.0)	0.135	9.0 (7.0; 11.0)	12.0 (9.0; 15.0)	<0.001
length of ICU stay (days)	0	4.1 (2.1; 7.6)	5.2 (2.9; 9.2)	1.8 (1.3; 4.5)	<0.001	4.7 (2.3; 8.2)	2.2 (1.5; 7.4)	0.014
ICU mortality	0	40 (19.4)	4 (2.9)	36 (53.7)	<0.001	0	40 (69.0)	
28-day mortality	0	54 (26.2)	15 (10.8)	39 (58.2)	<0.001	0	54 (93.1)	
90-day mortality	0	58 (28.2)	17 (12.2)	41 (61.2)	<0.001	0	58 (100)	

Continuous variables are summarized as medians with interquartile ranges (IQR), categorical variables as counts and percentages.
Organ dysfunction was defined as unchanged or worsening SOFA score or death within 7 days in intensive care unit.
Abbreviations: SOFA, Sequential Organ Failure Assessment; SOFA-improvers, patients whose SOFA score decreased during the 7 day follow-up; SOFA-non-improvers, patients whose SOFA score stayed the same or increased, or who died during the 7-day follow-up; Chronic Kidney Disease defined as glomerular filtration rate (GFR) <60 ml/minute/1.73 m2 or chronic dialysis at least one week prior to ICU admission; APACHE, Acute Physiology and Chronic Health Evaluation; Sepsis and septic shock defined according to Sepsis-3 [17]; AKI, Acute Kidney Injury, defined by Kidney Disease Improving Global Outcomes (KDIGO) Clinical Practice Guidelines [18]; RRT, Renal Replacement Therapy; CRRT, Continuous Renal Replacement Therapy; IHD, Intermittent Hemodialysis; SAPS II, Simplified Acute Physiology Score; ICU, Intensive Care Unit.

Table S10 Results of univariable analyses for organ dysfunction and 90-day mortality for sepsis sub-group

		Organ dysfunction			90-day mortality		
	missing n	SOFA-improvers n=139	SOFA-non-improvers n=67	p	Survivors n=148	Non-survivors n=58	p
SOFA -score[a]	1	9.0 (7.0; 12.0)	10.0 (7.0; 13.0)	0.135	9.0 (7.0; 11.0)	12.0 (9.0; 15.0)	<0.001
SAPS II[a]	2	43.0 (36.0; 56.3)	54.5 (37.5; 67.0)	0.011	41.0 (34.0; 52.0	61.0 (51.0; 72.0)	<0.001
APACHE[a]	2	23.0 (18.0; 29.0)	27.0 (20.0; 34.3)	0.013	22.0 (18.0; 28.0)	30.0 (24.0; 35.0)	<0.001
Mean arterial pressure (mmHg)[b]	2	74.1 (70.1; 78.2)	69.0 (62.5; 73.0)	<0.001	73.8 (69.9; 77.7)	69.0 (61.8; 72.7)	<0.001
Heart rate (bpm)[b]	2	90.8 (80.4; 102.5)	96.1 (80.5; 107.9)	0.281	89.8 (79.3; 101.9)	96.7 (83.3; 108.4)	0.022
Norepinephrine dose (µg/kg/min)[b]	3	0.10 (0.05; 0.23)	0.23 (0.08; 0.44)	<0.001	0.10 (0.04; 0.22)	0.25 (0.13; 0.49)	<0.001
Arterial lactate (mmol/l)[b]	9	1.5 (1.0; 2.4)	3.0 (1.4; 5.8)	<0.001	1.4 (1.0; 2.3)	4.2 (2.0; 6.3)	<0.001
Capillary refill time (s)[b]	0	4.5 (2.7;6.9)	5.6 (3.1; 13.1)	0.037	4.2 (2.5; 6.6)	9.4 (3.9; 15.4)	<0.001
Mottling score[b]	0	0.0 (0.0; 0.3)	0.0 (0.0; 0.6)	0.110	0.0 (0.0; 0.1)	0.4 (0.0; 1.6)	<0.001
Peripheral temperature (°C)[b]	1	33.0 (31.8; 34.1)	32.4 (30.9; 33.8)	0.055	32.9 (31.7; 34.1)	32.5 (31.1; 33.8)	0.062
Central to peripheral temperature gradient (°C)[b]	4	4.1 (2.7; 5.2)	4.0 (2.4; 5.4)	0.964	4.0 (2.7; 5.2)	4.0 (2.5; 5.4)	0786
Mean Peripheral StO_2 (%)[b]	1	67.8 (56.6; 74.1)	64.8 (51.8; 71.6)	0.080	68.8 (57.5; 74.8)	62.0 (49.6; 67.8)	<0.001
AUT below StO_2 thresholds (%min)[b]							
60 %	0	1904.5 (13.8; 12820.8)	1426.8 (13.5; 14506.5)	0.753	1001.1 (1.2; 10912.0)	3535.3 (498.9; 21756.1)	0.004
50 %	0	66.5 (0.0; 2587.5)	70.3 (0.0; 3579.0)	0.781	19.5 (0.0; 2031.7)	571.1 (0.0; 6220.5)	0.012
40 %	0	0.0 (0.0; 292.8)	0.0 (0.0; 856.5)	0.484	0.0 (0.0; 241.0)	16.5 (0.0; 1156.1)	0.014
AOT above StO_2 80 % (%min)[b]	0	58.0 (0.0; 1494.0)	0.0 (0.0; 66.3)	0.001	63.0 (0.0; 1860.1)	0.0 (0.0; 22.8)	<0.001
Time below StO_2 thresholds (min)[b]							
60 %	1	227.0 (6.5; 1432.0)	308.0 (8.8; 1145.0)	0.941	136.8 (1.1; 1292.4)	609.5 (117.5; 1648.0)	0.012
50 %	1	7.5 (0.0; 346.5)	21.0 (0.0; 555.5)	0.883	3.0 (0.0; 319.1)	134.5 (0.0; 734.5)	0.020
40 %	1	0.0 (0.0; 28.0)	0.0 (0.0; 91.8)	0.571	0.0 (0.0; 15.3)	3.0 (0.0; 148.3)	0.009
Time above StO_2 80 % (min)[b]	1	15.5 (0.0; 460.0)	0.0 (0.0; 47.2)	0.002	19.8 (0.0; 469.5)	0.0 (0.0; 11.0)	<0.001

All continuous parameters are summarized as medians and interquartile ranges (IQR).
Organ dysfunction was defined as unchanged or worsening SOFA score or death within 7 days in intensive care unit.
Abbreviations: StO2, tissue oxygen saturation; SOFA, Sequential Organ Failure Assessment; h, hour; SOFA; improvers, patients whose SOFA score decreased during the 7 day follow-up; SOFA-non-improvers, patients whose SOFA score stayed the same or increased, or who died during the 7-day follow-up; SAPS II, Simplified Acute Physiology Score; APACHE, Acute Physiology and Chronic Health Evaluation II; mmHg, millimeters of mercury; bpm, beats per minute; µg/kg/min, micrograms per kilogram per minute; mmol/l, millimoles per liter; s, seconds; °C, degrees of Celsius; AUT, area under StO_2 threshold; AOT, area over StO_2 threshold; min, minutes.
[a] 0-24 hours
[b] 48-hour mean

Table S11 Results of logistic regression models for organ dysfunction and 90-day mortality for sepsis sub-group

	Organ dysfunction		90-day mortality	
**Variables in the model**	**Odds ratio (95% Confidence interval)**	**p**	**Odds ratio (95% Confidence interval)**	**p**
**MODEL 1**				
Capillary refill time[a]	-	ns	-	ns
Chronic kidney disease	-	ns	-	ns
Lactate[a]	1.30 (1.09-1.56)	0.004	1.42 (1.18-1.72)	<0.001
Mean arterial pressure[a]	0.91 (0.86-0.97)	0.003	-	ns
Norepinephrine dose[a]	-	ns	-	ns
Peripheral StO_2[a]	-	ns	0.96 (0.93-1.00)	0.023
SAPS II score[b]	-	ns	1.06 (1.03-1.09)	<0.001
Smoker (ever)	2.3 (1.33-4.68)	0.021	-	ns
**MODEL 2**				
Capillary refill time[a]	-	ns	-	ns
Chronic kidney disease	-	ns	-	ns
Lactate[a]	1.31 (1.09-1.58)	0.003	1.36 (1.14-1.62)	<0.001
Mean arterial pressure[a]	0.91 (0.85-0.97)	0.003	-	ns
Mottling score[a]	-	ns	1.89 (1.09-3.28)	0.024
Norepinephrine dose[a]	-	ns	-	ns
SAPS II score[b]	-	ns	1.06 (1.02-1.09)	<0.001
Smoker (ever)	2.25 (1.11-4.57)	0.024	-	ns

Binary logistic regression models (forward stepwise, conditional). Model 1 contains peripheral 48-hour mean StO_2 and Model 2 contains 48-hour mean mottling score instead.
Organ dysfunction was defined as unchanged or worsening SOFA score or death within 7 days in intensive care unit.
Chronic kidney disease (moderate or severe) was defined as glomerular filtration rate <60 ml/minute/1.73 m^2 or chronic dialysis at least one week prior to ICU admission.
Abbreviations: SAPS II, Simplified Acute Physiology Score; MAP, Mean Arterial Pressure; StO_2, Tissue Oxygen Saturation; ns, not significant
[a] 48-hour mean
[b] 0-24 hours

Table S12 Reported problems in NIRS-monitoring.

Problem	Missing n	All patients<br>n=256
**Detachment of the cords**	16	21/240 (8.8%)
**Detachment of the probes**	16	17/240 (7.1%)
**Monitor related problems**	16	4/240 (1.7%)
**Problems with patient's skin**	16	3/240 (1.3%)
**Problems with patient positioning**	16	2/240 (0.8%)
**Problems with patient mobilization**	16	1/240 (0.4%)
**Other**	16	6/240 (2.5%)

References

1. Fliser D, Laville M, Covic A, Fouque D, Vanholder R, Juillard L, Van Biesen W. A European Renal Best Practice (ERBP) position statement on the Kidney Disease Improving Global Outcomes (KDIGO) clinical practice guidelines on acute kidney injury: part 1: definitions, conservative management and contrast-induced nephropathy. Nephrol Dial Transplant. 2012;27(12):4263-4272.
