# Supplementary figures and images for: Clinical outcomes and peripheral tissue oxygen saturation monitoring of the knee region by near-infrared spectroscopy in circulatory shock: a prospective observational cohort study
Source: Crit Care. 2025 Mar 19;29:125. doi: 10.1186/s13054-025-05363-1 (PMC11924835; doi:10.1186/s13054-025-05363-1)

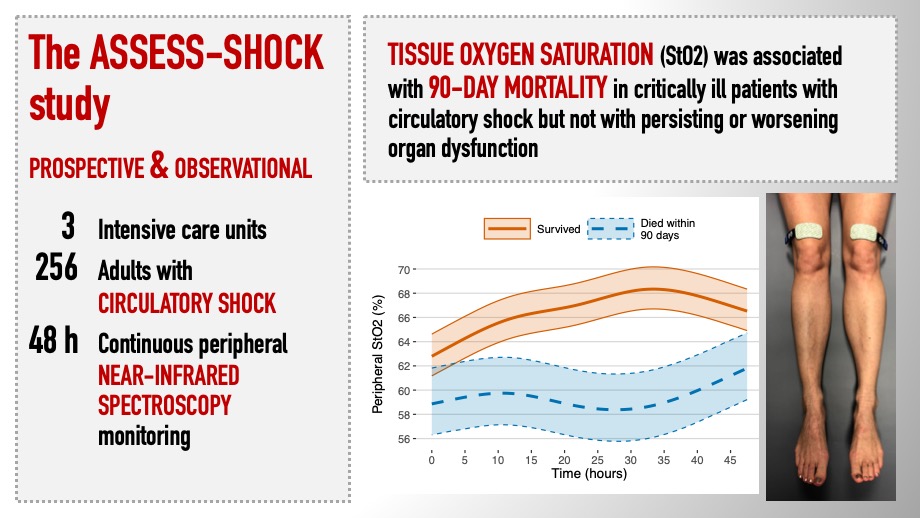

Supplement: Supplementary file 2 — Additional file2 (JPG 131 kb) [file 13054_2025_5363_MOESM2_ESM.jpg]
